# The IRE1α/XBP1s Pathway Is Essential for the Glucose Response and Protection of β Cells

**DOI:** 10.1371/journal.pbio.1002277

**Published:** 2015-10-15

**Authors:** Justin R. Hassler, Donalyn L. Scheuner, Shiyu Wang, Jaeseok Han, Vamsi K. Kodali, Philip Li, Julie Nguyen, Jenny S. George, Cory Davis, Shengyang P. Wu, Yongsheng Bai, Maureen Sartor, James Cavalcoli, Harmeet Malhi, Gregory Baudouin, Yaoyang Zhang, John R. Yates III, Pamela Itkin-Ansari, Niels Volkmann, Randal J. Kaufman

**Affiliations:** 1 Degenerative Diseases Program, Sanford Burnham Prebys Medical Discovery Institute, La Jolla, California, United States of America; 2 Department of Biological Chemistry, University of Michigan Medical Center, Ann Arbor, Michigan, United States of America; 3 Lilly Research Laboratories, Eli Lilly & Company, Lilly Corporate Center, Indianapolis, Indiana, United States of America; 4 Department of Internal Medicine, University of Michigan Medical Center, Ann Arbor, Michigan, United States of America; 5 NCIBI Department of Bioinformatics, University of Michigan Medical Center, Ann Arbor, Michigan, United States of America; 6 Department of Biology, Indiana State University, Terre Haute, Indiana, United States of America; 7 Department of Chemical Physiology and Cell Biology, The Scripps Research Institute, La Jolla, California, United States of America; University of Cambridge, UNITED KINGDOM

## Abstract

Although glucose uniquely stimulates proinsulin biosynthesis in β cells, surprisingly little is known of the underlying mechanism(s). Here, we demonstrate that glucose activates the unfolded protein response transducer inositol-requiring enzyme 1 alpha (IRE1α) to initiate X-box-binding protein 1 (*Xbp1*) mRNA splicing in adult primary β cells. Using mRNA sequencing (mRNA-Seq), we show that unconventional *Xbp1* mRNA splicing is required to increase and decrease the expression of several hundred mRNAs encoding functions that expand the protein secretory capacity for increased insulin production and protect from oxidative damage, respectively. At 2 wk after tamoxifen-mediated *Ire1α* deletion, mice develop hyperglycemia and hypoinsulinemia, due to defective β cell function that was exacerbated upon feeding and glucose stimulation. Although previous reports suggest IRE1α degrades insulin mRNAs, *Ire1α* deletion did not alter insulin mRNA expression either in the presence or absence of glucose stimulation. Instead, β cell failure upon *Ire1α* deletion was primarily due to reduced proinsulin mRNA translation primarily because of defective glucose-stimulated induction of a dozen genes required for the signal recognition particle (SRP), SRP receptors, the translocon, the signal peptidase complex, and over 100 other genes with many other intracellular functions. In contrast, *Ire1α* deletion in β cells increased the expression of over 300 mRNAs encoding functions that cause inflammation and oxidative stress, yet only a few of these accumulated during high glucose. Antioxidant treatment significantly reduced glucose intolerance and markers of inflammation and oxidative stress in mice with β cell-specific *Ire1α* deletion. The results demonstrate that glucose activates IRE1α-mediated *Xbp1* splicing to expand the secretory capacity of the β cell for increased proinsulin synthesis and to limit oxidative stress that leads to β cell failure.

## Abstract 2

Each time one eats, the endoplasmic reticulum of the pancreatic beta cells is forced to produce more insulin, thereby activating the IRE1α branch of the unfolded protein response pathway.

## Author Summary

One of the most remarkable features of the pancreatic beta cells—those that produce and secrete insulin to regulate glucose homeostasis—is their capacity to increase the synthesis of proinsulin (the insulin precursor) up to 10-fold after glucose stimulation. This dramatic increase in the synthesis of proinsulin is a challenge to the proximal secretory pathway and triggers an adaptive stress response, the unfolded protein response, which is coordinated by the IRE1α enzyme and the X-box-binding protein 1 (XBP1) transcription factor. Deletion of IRE1α specifically from the pancreatic beta cells in adult mice resulted in overt diabetic phenotypes such as high blood glucose. mRNA analysis revealed several hundred genes whose expression was coordinately regulated by glucose and IRE1α and whose functions are important for the beta cell secretory pathway. Furthermore, IRE1α also regulates the expression of mRNAs involved in the production of reactive oxygen species (ROS), and we could show that, in fact, oxidative stress is a primary mechanism that causes beta cell failure upon collapse of the secretory pathway. Finally, in experiments with murine and human islets (the regions of the pancreas where secretory beta cells are located), we observed that while IRE1α does not regulate the expression of the gene encoding insulin, it determines final insulin levels by controlling translation of proinsulin mRNA.

## Introduction

Type 2 diabetes (T2D) is a disease epidemic caused by failure of β cells to produce sufficient insulin to maintain glucose homeostasis [1]. In response to obesity, insulin resistance and hyperglycemia pressure β cells to increase preproinsulin synthesis, processing, and secretion. Although β cells can compensate by increasing insulin production, approximately one-third of individuals with insulin resistance eventually develop β cell failure and diabetes [2]. Unfortunately, the mechanisms leading to β cell failure in T2D are poorly understood, although factors include genetic lesions, hyperglycemia, hyperlipidemia, and inflammatory cytokines [3]. The β cell, unlike other professional secretory cells, is uniquely specialized for glucose-stimulated insulin secretion (GSIS) in order to respond to daily fluctuations in blood glucose. Upon glucose-stimulated release of insulin granules, preproinsulin mRNA translation increases up to 10-fold [4–6]. Since glucose has a modest short-term effect on insulin gene transcription [7,8], it is surprising how little is known of the underlying mechanism(s) of glucose-stimulated insulin mRNA translation and recruitment to the endoplasmic reticulum (ER), which represents the earliest rate-limiting step in insulin biosynthesis. For the β cell to accommodate increased preproinsulin synthesis, it is necessary to expand the secretory pathway for preproinsulin cotranslational translocation, folding, processing, trafficking, and storage in secretory granules. Recent studies suggest that increased proinsulin synthesis overwhelms the capacity of the ER to properly fold, process, and secrete insulin in response to glucose and activates the unfolded protein response (UPR) [3,9–12].

The UPR is an adaptive mechanism to prevent accumulation of misfolded protein in the ER [13,14]. Inositol-requiring enzyme 1α (IRE1α) is the most conserved transducer of the UPR that signals through initiating unconventional splicing of X-box-binding protein 1 (*Xbp1)* mRNA. Cytosolic splicing of *Xbp1* mRNA removes 26 nucleotides to create a translational frame shift that produces a potent basic-leucine zipper-containing (bZIP) transcription factor (TF) (XBP1s) that induces genes encoding functions within the ER, including protein synthesis, folding, and trafficking, N-linked glycosylation, lipid biosynthesis, and ER-associated protein degradation (ERAD) [14–16], while mRNAs inhibited by XBP1s or induced by unspliced XBP1u are mercurial. In addition, the endoribonuclease (RNase) activity of IRE1α degrades its own mRNA [17], as well as additional mRNAs containing CUGCAG or similar RNA recognition motifs in a process termed regulated IRE1α-dependent degradation (RIDD), in theory to reduce the ER protein-folding burden [18,19]. Further complicating the pathway is the recent function attributed to IRE1α’s RNase activity in microRNA (miRNA) biogenesis and/or degradation; however, these endonucleolytic targets are not conserved in all eukaryotic cell types and are not as essential for cell function as IRE1α-mediated cytosolic splicing of *Xbp1* mRNA [20–22]. In metazoans, the UPR signals through two additional ER transmembrane sensors, the PKR-like ER kinase (PERK) and the bZIP TF activating transcription factor 6 α (ATF6α), where only the latter is dispensable for organismal survival and β cell function [3,9–12,23–30].

A physiological requirement for IRE1α/XBP1s in β cell function was suggested from analysis of Wolfram syndrome, also known as DIDMOAD (diabetes insipidus, diabetes mellitus, optic atrophy, and deafness), in which patients experience ~60% mortality by the age of 35 [31]. The Wolfram syndrome 1 (*Wfs1*) gene encodes an ER-resident protein associated with protein folding, calcium homeostasis, glucose-stimulated cAMP production, and degradation with ATF6α [32,33]. As XBP1s activates the *Wfs1* promoter [34], the IRE1α-XBP1s-WFS1 pathway represents a direct link between protein folding in the ER, the UPR, β cell failure, and a diabetic patient cohort.

Previous studies on IRE1α function in β cells have not measured the effects deletion of *Ire1α* in adult differentiated β cells [35–37]. β cell-specific, embryonic deletion of *Xbp1* caused hyperactivation of IRE1α RNase to degrade mRNAs encoding proinsulin processing enzymes;prohormone convertases 1 and 2 (PC1 and PC2) and carboxypeptidase E (CPE), leading to the conclusion that IRE1α/XBP1 is required for proinsulin to insulin maturation [38]. In the context of these results, we sought to measure the importance of IRE1α signaling in differentiated primary β cells. Therefore, we employed inducible deletion of *Ire1α* in mature β cells and massive parrallel sequencing to uncover mRNAs altered upon glucose stimulation in an IRE1α-dependent manner. We then compared our results with previously reported IRE1α/XBP1s and IRE1α/RIDD targets to identify the novel and overlapping changes in mRNAs that are IRE1α-dependent in glucose-stimulated islets. Our results reveal that glucose-inducible, IRE1α-dependent mRNAs encode numerous functions important for the β cell secretory pathway, including ribosome recruitment to the ER, cotranslational translocation, signal peptide cleavage, protein folding, and trafficking, all of which are required for proper glucose-stimulated preproinsulin biosynthesis and conversion of preproinsulin to proinsulin. Indeed, in the absence of IRE1α, there is a defect in translation of proinsulin mRNA. However, mRNA-Seq also revealed many uncharacterized and unexpected mRNAs with diverse non-ER functions that are also dependent on IRE1α and glucose stimulation. In contrast, deletion of IRE1α in differentiated β cells increased expression of mRNAs encoding enzymes that produce reactive oxygen species (ROS), proteins of the plasma membrane, and extracellular matrix (ECM) that at least partially account for the oxidative stress, inflammation, and fibrosis measured in adult *Ire1α*-null islets. Furthermore, we show that oxidative stress is a primary mechanism that causes β cell failure upon collapse of the secretory pathway.

## Results

### *Ire1α* Deletion in Adult β Cells Decreases Proinsulin Biosynthesis at the Post-transcriptional Level

**Fig 1:**
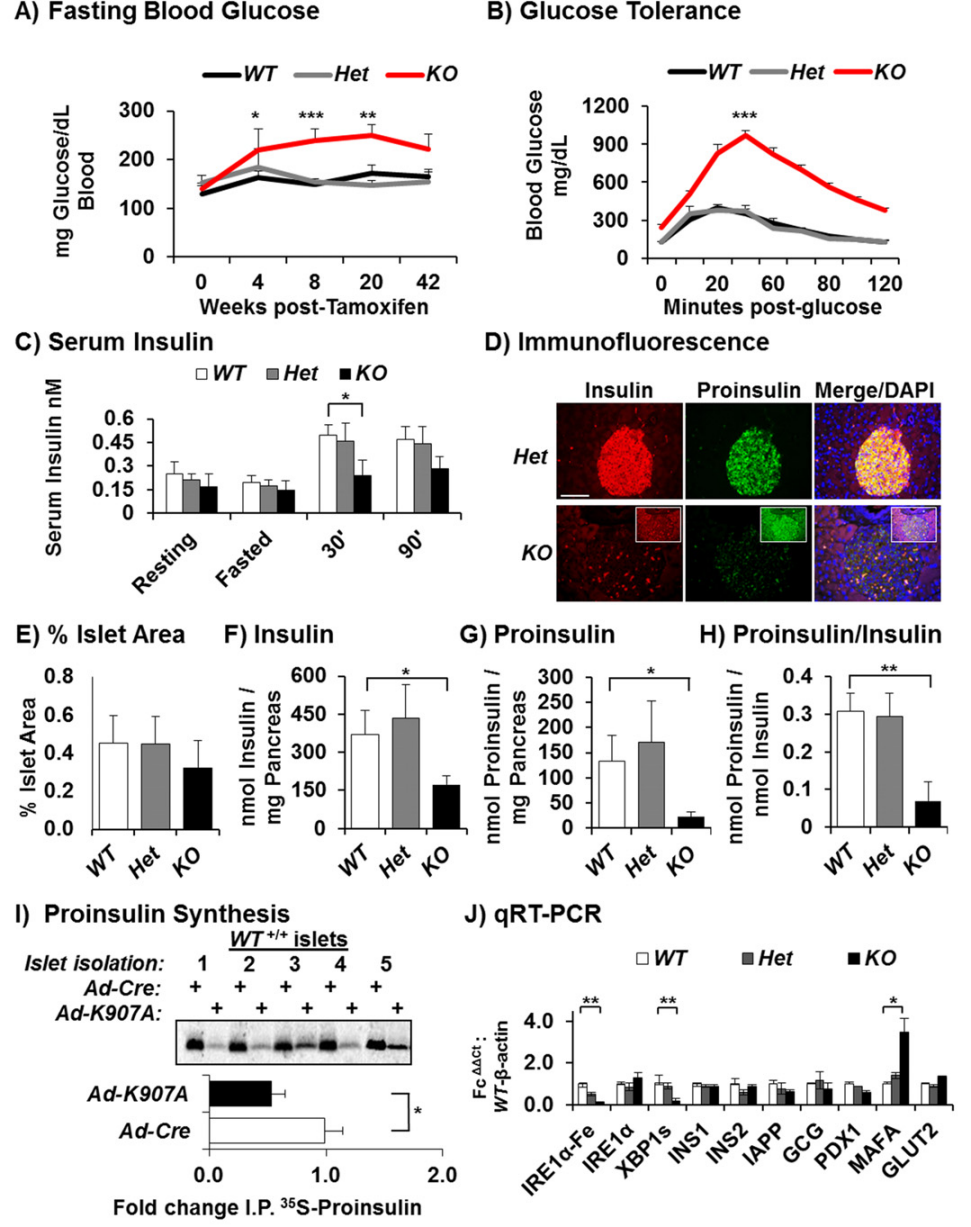
Tam-induced *Ire1α* deletion in adult β cells reduces proinsulin synthesis, insulin content, and insulin secretion, without altering insulin mRNA levels. (A) Blood glucose levels for 16-wk-old male mice following 4 h of fasting with increasing weeks post-Tam. Respectively for 4, 8, and 20 wk post-Tam ([*p* = 0.042, 0.009, 0.031], [*WT* ^*Fe/+*^ *n* = 8, *KO* ^*Fe/-; Cre*^ *n* = 10]). (B) Glucose tolerance tests (GTTs) performed on 16-wk-old male mice at 6 wk post-Tam and the areas under the curves 6 wk post-Tam. The values for statistical significance in Fig 1A and S1A Fig were calculated from areas under the GTT curves. The data and statistics for the GTTs and all other data except when indicated are within S1 Data. ([*WT* ^*Fe/+*^, *Het-I* ^*Fe/+; Cre*^, *Het-B* ^*Fe/-*^ and *KO* ^*Fe/-; Cre*^ *n* = 8], [*p* = 1.408 x 10^−7^, *WT* ^*Fe/+*^ versus *KO* ^Fe/-; Cre^]). (C) Serum insulin levels in mice 6 wk post-Tam: fed, 4 h fasted, 30 and 90 min after refeeding ([*n* = 4, all groups], student’s *t* test for significance for *WT* ^*Fe/+*^ versus *KO* ^*Fe/-; Cre*^ [*p* = 0.044]). (D) Immunofluorescence microscopy of islets co-stained for insulin (red), proinsulin (green), and DAPI (blue); see S3B Fig for additional examples. Scale bar, 100 μm. (E) Percent islet areas were determined on 6-wk post-Tam pancreas by outlining 138, 153, 234, and 297 cross sections from 9, 9, 11, and 14 mice *WT* ^*Fe/+*^, *Het-I* ^*Fe/+; Cre*^, and *KO* ^*Fe/-; Cre*^ groups, respectively. (F and G) Insulin and proinsulin ELISAs of acid ethanol extracts from 6 wk post-Tam mouse pancreas (*WT* ^*Fe/+*^ versus *KO* ^*Fe/-; Cre*^; [insulin, *p* = 0.039, proinsulin, *p* = 0.031], [*n* = 5, all groups]). (H) Individual mouse proinsulin/insulin ratios were determined and averaged ([*p* = 0.009], [*n* = 5]). (I) Islets were shifted from 4 mM to 25 mM glucose for 30 min in [^35^S]-Cys/Met in order to determine the synthesis rate during high glucose by antiproinsulin immunoprecipitation IP ([*n* = 3] for *WT* ^*Fe/+*^, *Het-I* ^*Fe/+; Cre*^, and *KO* ^*Fe/-; Cre*^), ([*n* = 6] for *+/+* infected with *Ad-Cre* versus *Ad-ΔR* (*p* = 0.019)]. Since limiting amounts of a homemade proinsulin antibody was used for the first five lanes, the Ad experiments used a commercial antibody that produced consistent results. (J) Real-time PCR (quantitative real-time PCR [qRT-PCR]) of total RNA isolated from islets at 6 wk post-Tam ([*n* = 5], [*p* = 0.022**, 0.039*, and 0.047*]) for *Ire1α* deletion-specific, *Xbp1* spliced-specific, and *Mafa* mRNAs, respectively.

To study the function of IRE1α in differentiated β cells and since *Ire1α* deletion causes embryonic lethality, we analyzed the requirement for IRE1α in β cells by generating and characterizing mice with one floxed *Ire1α* ^*Fe*^ allele [39] in combination with either a wild-type (*WT*) *Ire1α* ^+^ or an *Ire1α—* null allele [40]. Deletion of the floxed *Ire1α* ^*Fe*^ allele is mediated by a Cre recombinase-estrogen receptor fusion protein driven by the rat *Ins2* promoter, which is expressed in pancreatic β cells from midgestation (E9–E11.5) and activated by tamoxifen (Tam) administration [41]. This cross yields *Ire1α* ^*Fe/-; Cre+*^ (herein designated *KO*) mice at nearly the expected frequency as the *Ire1α* ^*Fe/+; Cre-*^ *WT*, *Ire1α* ^*Fe/-; Cre-*^ (full-body heterozygous at birth; *Het-B*) and *Ire1α* ^*Fe/+; Cre+*^ (Tam-induced β cell-specific heterozygous; *Het-I*) littermate controls. This allows more efficient *Ire1α* deletion as only one floxed allele requires deletion and provides two different heterozygous genotypes for comparison to the *WT* and *KO* groups. Undeleted *KO* mice exhibit normal blood glycemia; however, glucose intolerance became significant at 2 wk post-Tam injection and peaked at 6 wk (Fig 1A and 1B and S1A Fig). A similar diabetic phenotype was observed upon analysis of Tam-induced deletion of homozygous floxed mice *Ire1α* ^*Fe/Fe; Cre+*^ (*KO*) (S1A Fig). Only the *KO* mice displayed significantly reduced levels of insulin and proinsulin within the serum, pancreas, and islets compared to the heterozygous or *WT* mice (Fig 1A–1I and S1A–S1C Fig). Peak glucose intolerance for the *KO* mice occurred 6 wk post-Tam when *Ire1α* deletion was most efficient (Fig 1A and 1J and S1A Fig); therefore, most islet experiments were conducted at this time, unless otherwise noted. Compared to the developmental deletion models of *Ire1α* and *Xbp1* previously reported [35–37,42], Tam-induced *Ire1α* deletion in β cells of adult mice caused a significantly greater diabetic phenotype (Fig 1A–1I and S1A–S1C Fig). The decreased basal serum insulin in the *KO* mice became more pronounced after a fast and refeed, indicating a defect in postprandial insulin secretion (Fig 1C). Consistent with β cell failure, the *KO* mice also exhibited higher levels of serum proinsulin (S1B Fig). Significantly, although the percent islet mass was slightly reduced in the *KO* mice, this decrease alone could not account for the diabetic phenotype (Fig 1E). A more accurate measure of total insulin and proinsulin content in pancreas extracts by ELISA showed a significant reduction in both insulin and proinsulin in the *KO* group, with proinsulin being more reduced than insulin (Fig 1F–1H). To determine whether the reduced pancreatic proinsulin content was accompanied by a defect in proinsulin synthesis, isolated islets were radiolabeled for 30 min under high glucose with [^35^S]-Cys/Met. Compared to *WT* and heterozygous islets, proinsulin synthesis was significantly reduced in the islets from *KO* mice (S1 Data). In addition, infection of *WT* islets with adenoviruses that express either Cre as control (*Ad-Cre*) or a dominant-negative IRE1α-K907A mutant (*Ad-ΔR*) demonstrated only the latter selectively reduced proinsulin synthesis (Fig 1I and S1 Data). Taken together, the slightly reduced islet area coupled with the reduced proinsulin synthesis accounts for the reduced pancreatic insulin and proinsulin contents and abnormal glucose homeostasis in the *KO* mice (Fig 1A–1I and S1A–S1C Fig). In contrast to the beta cell-specific *Xbp1* deletion in which IRE1α RNase hyperactivation occurs [37], the mRNA levels encoding INS1 and INS2 and the proinsulin-processing enzymes—i.e., prohormone convertases (PC1, PC2) and CPE—were not significantly altered upon β cell-specific *Ire1α* deletion, despite an 88% decrease in the floxed *Ire1α* mRNA expression and an 81% decrease in *Xbp1* mRNA splicing (Figs 1J and 3A). In addition, reduced IRE1α in insulinoma cells was reported to also reduce proinsulin synthesis [35] and is consistent with our findings in islets. Importantly, these results show that adult β cells require functional IRE1α to maintain proinsulin mRNA translation, granule storage, and insulin secretion but not to maintain the expression of *Ins1*, *Ins2*, and most other β cell-specific mRNAs (Figs 1J and 3A).

In addition to the *Ad-Cre* control used on islets in vitro (Fig 1I), to further ensure the diabetic phenotype did not result from nonspecific expression of the *RIP-CreER* allele [43] and *Ire1α* deletion in another tissue in vivo, such as the hypothalamus [44], we measured levels of serum dopamine, which is synthesized in the arcuate nucleus of the hypothalamus. This analysis did not detect a significant difference between the *KO* and control mice (S1D Fig). In addition, although we detected Cre positive staining in brain sections, there was little difference in growth hormone-releasing hormone (GHRH) expression between the *KO* mice and the controls (S1E Fig). Finally, the *KO* islets and the *WT* islets expressing *Ad-ΔR* both demonstrated reduced proinsulin synthesis without affecting *Ins1* or *Ins2* mRNA levels in the *KO* (Fig 1I, S1 Data and Fig 1J). Therefore, we conclude that *Ire1α* deletion in β cells reduces proinsulin mRNA translation and is a primary molecular basis for the diabetic phenotype.

Generally, quantitative real-time PCR (qRT-PCR) of β cell-specific mRNAs did not detect a significant difference upon *Ire1α* deletion, although *Mafa* (v-maf avian musculoaponeurotic fibrosarcoma oncogene homolog A) mRNA expression was consistently increased within *KO* islets (Figs 1J and 3A). However, immunofluorescence microscopy demonstrated that the *WT* islets actually contained higher overall and nuclear MAFA protein than *KO* islets (S3A Fig), while PDX1 nuclear localization was not affected (S3A Fig). This is consistent with the finding that oxidative stress decreases nuclear localization of MAFA [45,46]. Although the peak of hyperglycemia occurred at 6 wk post-Tam injection, there was no significant increase in terminal deoxynucleotidyl transferase dUTP nick end labeling (TUNEL) positivity at this time or at 12 wk post-Tam injection. However, long after Tam-induced deletion (>6 mo), the glucose intolerance slightly improved, suggesting β cell recovery, possibly by expansion of nondeleted β cells and/or adaptation of the deleted cells (S2 Data). Therefore, the mechanism for the diabetic phenotype in the *KO* mice is not due to excessive β cell death but is most likely due to underlying XBP1s-dependent defects caused by *Ire1α* deletion in the β cell. Taken together, these results indicate IRE1α is required within the β cell for proinsulin mRNA translation, without significantly affecting insulin mRNA steady-state levels.

### *Ire1α* Deletion in β Cells Causes ER Stress

**Fig 2:**
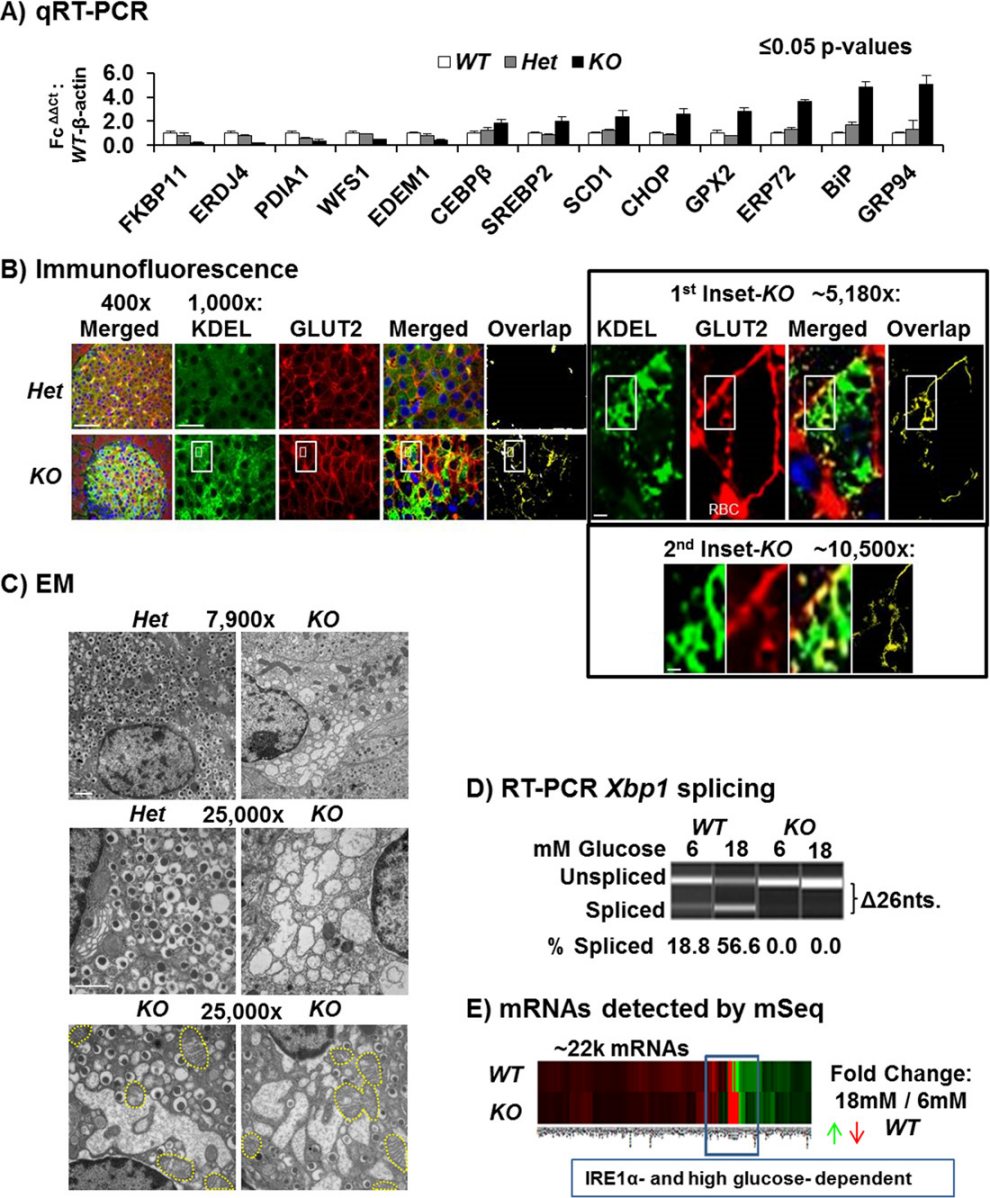
*KO* islets exhibit ER stress. (A) qRT-PCR of UPR genes in islets isolated 6 wk post-Tam and incubated in 11 mM glucose 16 h ([*n* = 5], [*p* ≤ 0.05]). (B) Immunofluorescence microscopy of pancreas sections stained for KDEL (BIP and GRP94) (green), the plasma membrane protein GLUT2 (red), and nuclei DAPI (blue). Overlap of red/green channels represents defective compartmentalization that was found to be increased in the *KO* ^*Fe/-; Cre*^ as shown in yellow. Scale bars, 400x = 50 μm, 1,000x = 10 μm, 5,180x = 2 μm and 10,500x = 1 μM. Additional examples are shown in S3B Fig. (C) EM of adult mouse (16 wk old) islets and their β cells from mice 2 wk post-Tam. Scale bars, both panels, 1 μm. Distended mitochondria are outlined with yellow dashes. (D) Conventional PCR flanking the 26 nt intron in *Xbp1* mRNA spliced by IRE1α from the islet complementary DNAs (cDNAs) used for mRNA-Seq analysis, 6 mM versus 18 mM glucose. Results representative of *n* = 5 per genotype. (E) Global heatmap for the ~22,000 mRNAs detected by mRNA-Seq for 18 mM *KO* ^*Fe/-; Cre*^ & *WT* ^*Fe/+*^ samples; green and red indicate increased and decreased expression. The blue box indicates genes with inverse expression dependent on IRE1α and high glucose.

Islets isolated from *KO* mice at 6 wk post-Tam injection demonstrated reduced expression of previously described XBP1s target genes *Atf6α*, *Pdia1*, *Fkbp11*, *Erdj4*, and *Wfs1* [34,39,47] and increased expression of ER chaperones *Hspa5* (immunoglobulin binding protein [BIP]), *Grp94*, and *Erp72* and proapoptotic *Ddit3* (Fig 2A). Immunofluorescence microscopy, qRT-PCR, and mRNA-Seq analyses also demonstrated increased BIP and GRP94 in *KO* islets (Fig 2A and 2B, S3B, S4A and S5C Figs). In addition, the *KO* β cells exhibited increased colocalization of the plasma membrane-resident protein GLUT2 with the KDEL-containing ER chaperones BIP and GRP94 (Fig 2B and S3B Fig), indicative of an ER-to-Golgi trafficking defect in *KO* β cells. Electron microscopy (EM) revealed that *KO* β cells contain many distended ER/Golgi membranes and/or empty vesicles, a 43% reduction in insulin granules, distended mitochondria, pyknotic nuclei, and multilamellar vesicles suggestive of autophagy (Fig 2C [yellow outlines] and S2A Fig, bottom). These dramatic morphological changes were not observed in Tam-induced *Het-I* control islets, indicating IRE1α is required to maintain organelle integrity in β cells. Next, we studied the effect of *Ire1α* deletion on glucose-regulated gene expression, by mRNA-Seq analysis on islets after 72 h incubation in 6 mM or 18 mM glucose to chronically stimulate insulin production and because 18mM glucose was reported to cause insulin mRNA degradation by IRE1α in insulinoma cells [48]. RT-PCR using primers flanking the unconventional intron spliced by IRE1α demonstrated treatment with 18 mM glucose increased *Xbp1* mRNA splicing by 56.6% in *WT* islets compared to undetectable levels in *KO* islets (Fig 2D). Similarly, qRT-PCR confirmed *Ire1α* deletion and *Xbp1* mRNA splicing were >90% decreased in *KO* islets (S3A Fig).

### mRNA-Seq Identifies IRE1α- and Glucose-Dependent mRNAs

**Fig 3:**
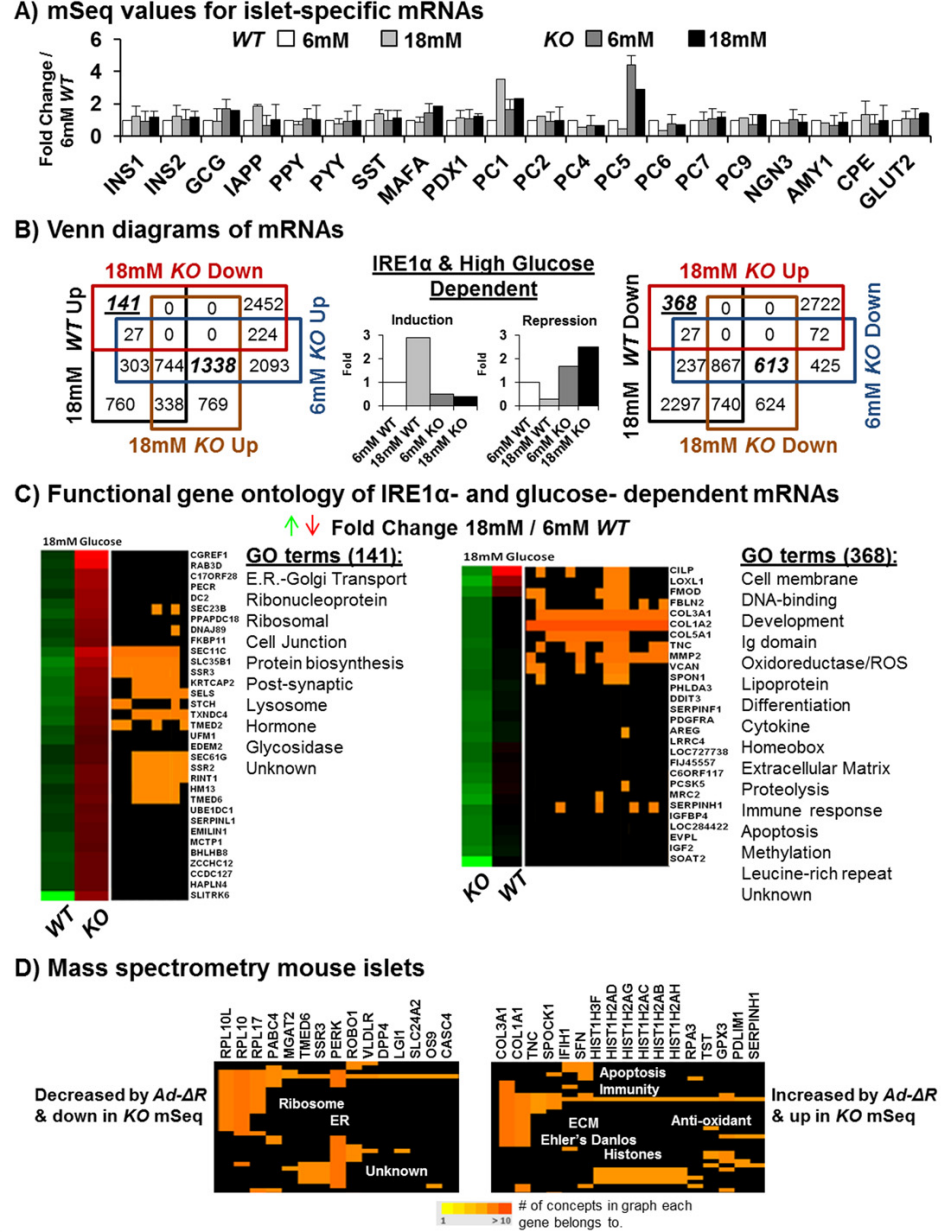
mRNA sequencing identifies IRE1α- and glucose-dependent mRNAs in islets. (A) mRNA-Seq data on β cell-specific mRNAs. The results show no significant change to INS1 or INS2 in the *KO* ^*Fe/-; Cre*^ samples, while MAFA, GCG, and PC5 are increased by deletion ([*n* = 5], [18 mM *KO* ^*Fe/-; Cre*^, *p*-values ≤ 0.05]). mRNA-Seq expression fold changes were normalized relative to the 6 mM *WT* ^*Fe/+*^ islet context. (B) Four-way Venn diagrams of *WT* ^*Fe/+*^ versus *KO* ^*Fe/-; Cre*^ islets during 6 mM versus 1 8mM glucose exposur*e* for 72 h. *Ire1α*-dependent mRNAs are in bold italics, while those also dependent on high glucose are in bold, italicized, and underlined font. At the center, bar graphs representing the *Ire1α*- and glucose-dependent trends of interest are labeled “Induction” and “Repression.” (C) Combined **DAVID** (the Database for Annotation, Visualization and Integrated Discovery) and “ConceptGen” GO analysis of *Ire1α-* and glucose-dependent mRNAs. Categories shown are specifically found in the genotype, while the shared categories have been omitted for simplicity, although no single mRNA was common between the groups. (D) Mass spectrometry of murine islets infected with *Ad-IREα-K907A (Ad-ΔR)* versus *Ad-β-Galactosidase* (*β-Gal*). Proteins with ≥5 unique peptides detected per protein increased or decreased upon infection in triplicate were analyzed for GO using ConceptGen and DAVID web resources (*n* = 3). The proteins shown (Fig 3D) exhibit the same expression dependence for IRE1α as measured by mRNA-Seq (S2 Data).

**Fig 4:**
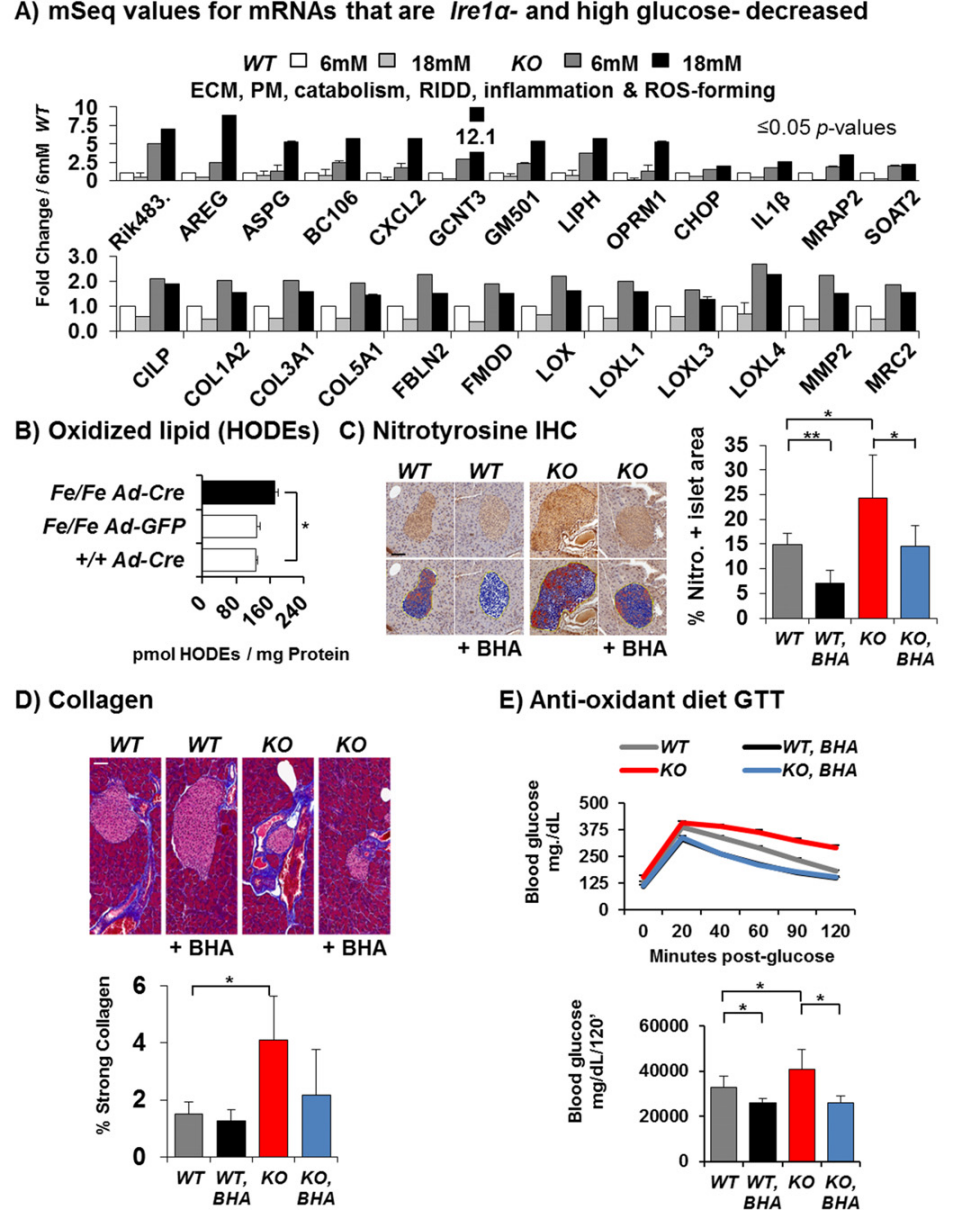
*KO* islets accumulate oxidative stress, inflammation, and fibrosis. (A) mRNA-Seq expression values for 25/368 of the mRNAs identified by Venn analysis (Fig 3C; right panel, underlined) that are reduced by *Ire1α* because of glucose that accumulates in the *KO* ^*Fe/-; Cre*^ ([*n* = 5], [*p*-values ≤ 0.05]). (B) Oxidized lipid (hydroxyl-octadecadienoic acids, HODEs) from islets of the indicated genotypes infected with *Ad-Cre Ad-GFP* or no virus control ([*n* = 2; controls versus *n* = 3; *Ad-Cre*], [*p* = 0.00434]). (C) Antinitrotyrosine immunohistochemistry (IHC) of islets from 8-mo-old *WT* ^*Fe/Fe*^ and *KO* ^*Fe/Fe; Cre*^ mice 15 wk post-Tam with or without BHA diet for 3 wk. (Scale bar, 50 μm) (*WT* ^*Fe/Fe*^ [*n* = 4 with BHA], [*n* = 5 regular chow]), (*KO* ^*Fe/Fe; Cre*^ [*n* = 5 with BHA], [*n* = 6 regular chow]). (*p* = 0.00698; *WT* ^*Fe/Fe*^ versus *WT* ^*Fe/Fe*^ with BHA), (*p* = 0.04018; *WT* ^*Fe/Fe*^ versus *KO* ^*Fe/Fe; Cre*^) and (*p* = 0.04420; *KO* ^*Fe/Fe; Cre*^ versus *KO* ^*Fe/Fe; Cre*^ with BHA). (D) Masson’s trichrome stain (blue) for collagens. Results demonstrate increased staining surrounding *KO* ^*Fe/Fe; Cre*^ islets with haemotoxylin (red) and eosin (black) co-stains. Quantification of percent strong collagen stain is shown below the images. Scale bar, 50 μm. (*WT* ^*Fe/Fe*^ [*n* = 4 with BHA], [*n* = 5 regular chow]), (*KO* ^*Fe/Fe; Cre*^ [*n* = 5 with BHA], [*n* = 6 without BHA]). Percent strong collagen stain significance for *WT* ^*Fe/Fe*^ without BHA versus *KO* ^*Fe/Fe; Cre*^ without BHA *p* = 0.01049). (E) 8-mo-old male mice carrying the doubly floxed allele (*Ire1α* ^*Fe/Fe*^ *)* with and without RIP-Cre 12 wk post-Tam had their pre-BHA GTTs taken, and then half were fed the antioxidant BHA supplemented chow diet for 3 wk or not before examining the mice by GTT again. (*WT* ^*Fe/Fe*^ [*n* = 11 with BHA], [*n* = 12 regular chow], [*p* = 0.035]), (*KO* ^*Fe/Fe; Cre*^ [*n* = 18 with BHA], [*n* = 16 without BHA], [*p* = 0.041]). *P*-values were calculated by one-tailed student’s *t* test comparison of the areas under the GTT curves for the biological replicates of control group *WT* ^*Fe/Fe*^ versus the Tam-induced *KO* ^*Fe/Fe; Cre*^ group.

mRNA-Seq on average detected >22,000 mRNAs in each sample that were visualized by heatmap (Fig 2E). mRNAs having altered expression with *p*-values ≤ 0.1 or ≤ 0.01 reduced the number to ~4,500 and ~1,700, respectively. Importantly, consistent with our previous results (Fig 1J), the levels of mRNAs encoding INS1 and INS2 were not significantly altered in *KO* islets as measured by both mRNA-Seq and qRT-PCR (Fig 3A and S4A Fig). In addition, as measured by qRT-PCR, mRNA-Seq also reported that mRNA expression of the XBP1s target genes *Wfs1*, *Atf6α*, *Edem1*, *Edem3*, and *Erdj4* was significantly reduced in *KO* islets, whereas the mRNA levels encoding BIP, GRP94, and DDIT3/CHOP were increased (Fig 2A, S4A and S5C Figs and S2 Data). Venn diagram analysis of the islet mRNA-Seq data was used to determine mRNAs that were *Ire1α* dependent, high glucose dependent, or dependent on both. The four-way Venn diagrams identified the expression of 613 decreased and 1,338 increased mRNAs in *KO* islets, of which 141 and 368 were simultaneously high glucose-dependent, respectively (Fig 3B and 3C and S2 Data). Among the 141 mRNAs that required both IRE1α and high glucose for induction, the most significant gene ontology (GO) terms included ER protein translocation, ER–Golgi protein transport machinery, ribosome and protein biosynthetic components, and the lysosome and glycosidases, as well as other intracellular processes with fewer representative mRNAs of each respective ontological group (Fig 3C, left). Strikingly, 76% of the 141 and 35% of the 368 IRE1α- and high glucose-dependent mRNAs have not yet been functionally characterized or previously associated with the IRE1α pathway (S3 Data). Similarly, only 22 of the 141 mRNAs were previously shown to bind XBP1 within their promoters (S4B Fig, left and S3 Data) [16]. In contrast to the mRNAs reduced upon *Ire1α* deletion, mRNA-Seq also uncovered 368 inversely regulated mRNAs that were increased in *Ire1α*-null islets and reduced during high glucose in *WT* islets (Fig 3B and 3C, right). These mRNAs encode proteins that are known RIDD targets, produce oxidative stress, and are involved in the ECM, plasma membrane, and immune cell signaling, as well as many other uncharacterized transcripts (Figs 3C and 4A, S4C and S5A Figs and S2 Data). The Venn diagram also compared the 368 inversely regulated mRNAs with previously identified XBP1s and RIDD targets (S4B and S4C Fig) [18,19]. These mRNAs increased by high glucose in *KO* islets but decreased in *WT* islets upon glucose stimulation could be (1) degraded by IRE1α, (2) repressed by XBP1s, (3) induced by XBP1u, (4) stabilized as a consequence of defective ribosome recruitment to the ER, (5) induced via alternative UPR pathways, (6) induced as a consequence of oxidative stress or inflammation (see below), or (7) derived from alternative cell types. To verify which changes in mRNA levels upon *Ire1α* deletion correlate with protein levels, isolated *WT* islets were infected in culture with adenoviruses expressing either β-Galactosidase (*Ad-β-Gal*) or the IRE1α K907A RNase mutant (*Ad-ΔR*), incubated in high glucose (18 mM), and analyzed by mass spectrometry after 72 h. Expression of the dominant-negative IRE1α caused a 21% reduction in insulin 1 and 2 peptides (S3 Data). The mass spectrometry analysis identified increases and decreases in proteins correlating with the mRNA changes detected by mRNA-Seq (Fig 3D and S3 Data).

### *Ire1α* Deletion Causes β Cell Failure Due to Oxidative Stress

The GO terms for those mRNAs that were increased by the absence of IRE1*α* include ROS-generating enzymes, such as inducible nitric oxide synthase (iNOS/NOS2), and the lysyl-oxidases (LOXs) that are involved in oxidation of collagens (COLs) that were also increased in the ECM (Fig 4A, S4C, S5A–S5C Figs). Consistent with increased oxidative stress, the *KO* islets also had significantly higher levels of mRNAs encoding glutathione peroxidases 2 and 3 (GPX2 and GPX3) and protein disulfide isomerases 4 and 5 (PDIA4 and PDIA5) (Fig 3D, S5A, S5C and S6A Figs). Therefore, we directly measured oxidative stress in *Ire1α*-deleted islets. Upon acute *Ad-Cre*-mediated *Ire1α* deletion in *Ire1α* ^*Fe/Fe*^ isolated islets, lipid peroxides (hydroxyl-octadecadienoic acids [HODEs]) increased 33% compared to *Ad-GFP*-infected or *Ad-Cre*-infected *Ire1α* ^*+/+*^ islet control groups (Fig 4B). In addition, increased nitrotyrosine staining was increased in *KO* pancreas sections after *Ire1α* deletion by Tam injection in vivo (Fig 4C). Similarly, tenascin C (TNC) mRNA and protein were increased in *KO* islets as measured by mRNA-Seq and mass spectrometry and then confirmed by immunohistochemistry (S5D Fig). Consistent with the increased collagen mRNA expression, Masson’s trichrome stain identified increased collagen staining surrounding the *KO* islets (Fig 4D and S5E Fig). Since previous studies suggested that antioxidant treatment reduces ER stress in β cells [9,49–51], we tested whether feeding mice chow supplemented with the antioxidant butylated hydroxyanisole (BHA) could improve β cell function upon *Ire1α* deletion. Therefore, at 12 wk post-Tam injection, mice were fed control chow or BHA-supplemented chow for 3 wk (12–15 wk post-Tam). Notably, feeding mice with BHA-supplemented chow significantly improved glucose homeostasis in mice with β cell-specific *Ire1α* deletion (Fig 4E) that was also reflected by decreased trends of nitrotyrosine, collagen, and TNC staining (Fig 4C and 4D, S5D and S5E Fig). Importantly, these findings indicate that *Ire1α* deletion causes β cell failure, at least in part, due to oxidative stress.

### Glucose Stimulates the IRE1α/XBP1s Pathway to Expand ER Capacity for Proinsulin Synthesis

**Fig 5:**
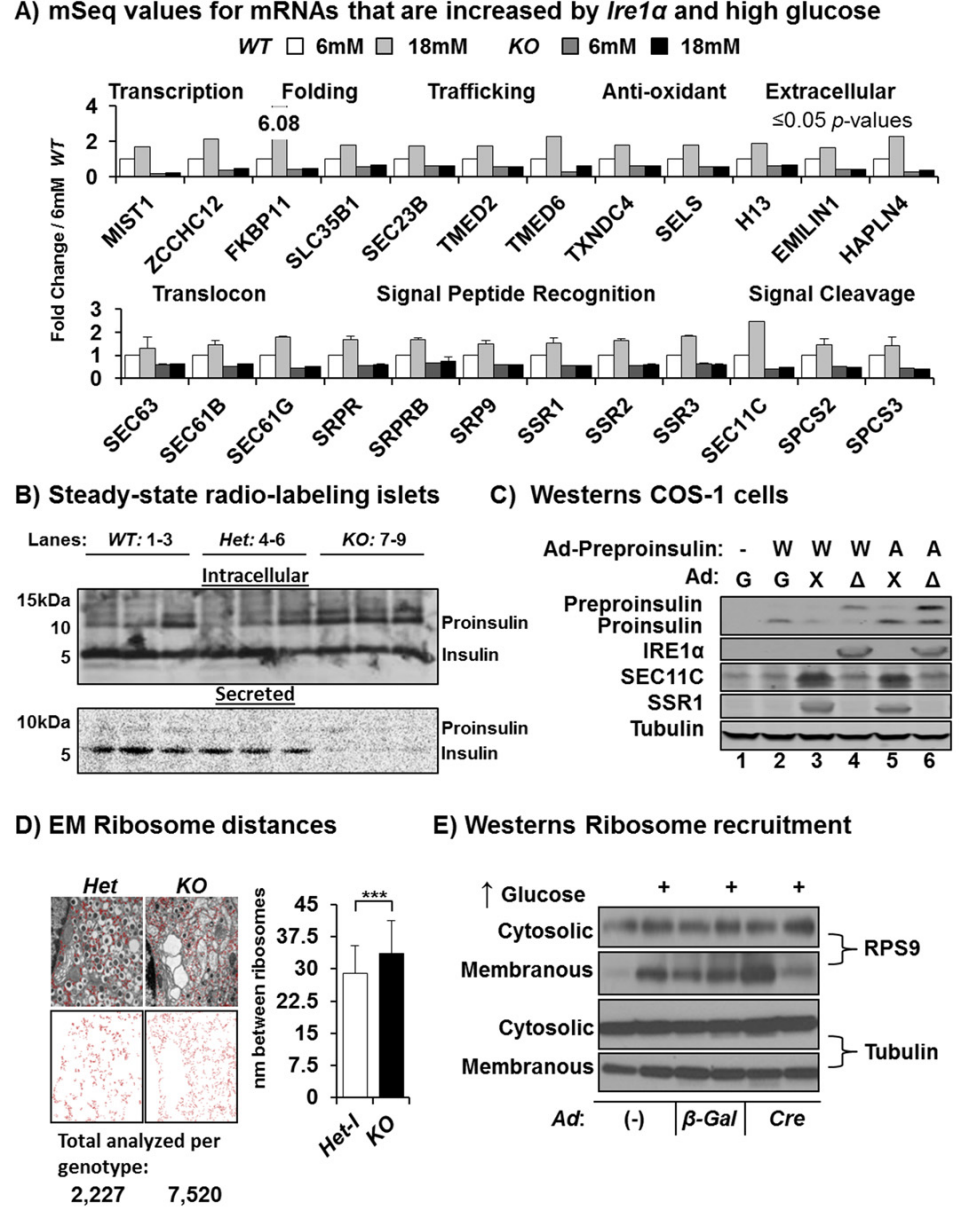
IRE1α mediated *Xbp1* splicing is necessary for proper signal peptide cleavage of preproinsulin and ribosome distribution. (A) mRNA-Seq expression values of 24/141 mRNAs identified (Fig 3B–3D) to be both *Ire1α-* and high glucose-dependent for their induction ([*n* = 5], [*p*-values ≤ 0.01]). (B) Autoradiograph from whole cell islet lysates prepared by steady-state (18 h) [^35^S]-Cys/Met radiolabeling from 12 wk post-Tam mice (*n* = 3) following peptide gel electrophoresis. (C) Western blotting for proinsulin/preproinsulin, IRE1α, SEC11C, SSR1, and tubulin after peptide gel electrophoresis of lysates prepared 72 h after COS-1 cells were coinfected with adenoviruses expressing *WT* preproinsulin, the Akita mutant (A), and/or the (G) GFP, (X) XBP1s, or (Δ) the dominant negative IRE1α-RNase mutant K907A representative results shown (*n* = 4). (D) Ribosomes and their relative position to one another were measured from electron micrographs at a magnification of 25,000x. Total numbers of ribosomes analyzed are shown below the images ([*n* = 3], [*p* = 2.2 x 10^−15^]). (E) Subcellular fractionation and western blot analysis for ribosomal small subunit 9 and tubulin of the *Ire1α* ^*Fe/Fe*^ β cell insulinoma line at after 2-h glucose shift from 12 mM to either 4 mM or 36 mM with or without infection by the indicated adenoviruses were blotted representative of (*n* = 3). The 12 mM condition western blots and the quantified results normalized to tubulin membranous/cytosolic are shown in S6D Fig.

Although the 141 mRNAs that require IRE1α for glucose induction encoded known functions in protein synthesis and the secretory pathway (Fig 3C, left, and Fig 5A, bottom), approximately half of these mRNAs have never been characterized or associated with the IRE1α/XBP1s pathway (Fig 3D and 3E (left panels), and S3 Data). Significantly, the most prominent GO cluster from the IRE1α-dependent and glucose-inducible group of 141 mRNAs included 12 with functions for SRP recruitment to the ER, translocon components, and the catalytic and structural subunits of the signal peptide cleavage complex (Fig 5A, bottom). These 12 mRNAs were induced up to 2.5-fold by glucose in an IRE1α-dependent manner, whereas their expression was reduced as much as 2.5-fold in the *KO* islets, i.e., ~4–6-fold difference during glucose stimulation, suggesting a major bottleneck in the signal peptide-dependent proximal secretory pathway of the β cell when compromised by *Ire1α* deletion.

In order to detect signal peptide cleavage in preproinsulin, which occurs very efficiently, special cellular contexts were utilized. Steady-state labeling demonstrated that, compared to control islets, the *KO* islets accumulated a slower migrating ~12 kDa species corresponding to the size of preproinsulin (Fig 5B and S1 Data). This steady-state labeling also showed decreased amounts of processed intracellular and secreted insulin and increased proinsulin that is likely due to the prolonged high glucose exposure in vitro (Fig 5B). To extend these findings and focus specifically on the preproinsulin-to-proinsulin processing step, signal peptide cleavage was analyzed in COS-1 cells that do not process proinsulin to insulin. Cells were coinfected with *Ad-preproinsulin* encoding either *WT* (W) or the *Akita* misfolded Cys96Tyr mutant (A) [52] and coexpressed with *Ad-GFP* (G) or *Ad-ΔR* (Δ). *Ad-preproinsulin* infection alone or in combination with *Ad-β-Gal* produced a single 10 kDa species representing proinsulin (Fig 5C, lanes 1–3). In contrast, coinfection of *Ad-preproinsulin* with *Ad-ΔR*, the IRE1α RNase-dead mutant incapable of splicing *Xbp1* mRNA, caused accumulation of a ~12 kDa insulin immune-reactive species, corresponding to preproinsulin, consistent with a defect in signal peptide cleavage when IRE1α-dependent *Xbp1* mRNA splicing is compromised (Fig 5C, lane 4), and this ~12 kDa species accumulated to a greater extent upon expression of the *Akita* proinsulin mutant (Fig 5C, lane 6). In addition, adenoviral overexpression of XBP1s was sufficient to induce SEC11C and signal sequence receptor 1 (SSR1) protein levels in COS-1 cells (Fig 5C). Because ribosome-membrane recruitment and translocon mRNAs were heavily *Ire1α*- and high glucose-dependent, we analyzed ribosome distribution in electron micrographs from *Het* and *KO* islets at 2 wk post-Tam injection. The *KO* had significantly more dispersed ribosomes, a hallmark of disordered polysomes, and more total monosome evidence for defect in polysome formation in the absence of IRE1α (Fig 5D) [53,54]. Consistent with this observation, *Ad-Cre* infection of an immortalized *Ire1α* ^*Fe/Fe*^ β cell line increased the monosome/polysome ratio (S6C Fig), further evidence of a translation initiation defect. Finally, we analyzed ribosome recruitment to the ER in the *Ire1α* ^*Fe/Fe*^ β cell line at 48 h after infection with *Ad-Cre* or controls. Cells were shifted from 12 mM glucose to media containing 4 mM, 12 mM, or 36 mM glucose for 2 h and then subjected to subcellular fractionation. Western blotting for the ribosomal small subunit proteins RPS9 and RPSA in cytosolic and membranous fractions from glucose-stimulated floxed *Ire1α* ^*Fe/Fe*^ insulinoma cells indicated *Ire1α* deletion in vitro disrupted glucose-stimulated recruitment of the ribosome to the membranous fraction (Fig 5E and S6D Fig). The increased monosomes detected in islets and the IRE1*α*-deleted insulinoma line (Fig 5D and S6C Fig) were accompanied by basally increased RPS9 and RPSA protein, but not increased mRNA levels (Fig 5E, S6D and S6E Fig). We next tested whether chemical inhibition of the IRE1α RNase activity in human islets affected *INS* mRNA levels after 24 h in low versus high glucose (S7A Fig). The chemical inhibition of IRE1α RNase overnight did not cause *INS* mRNA levels to accumulate but did block glucose-stimulated induction of proinsulin mRNA, supporting the hypothesis that XBP1s is needed for ER expansion to stimulate proinsulin mRNA translation (S7A Fig). The reduced levels of the XBP1s target gene *P58* ^*IPK*^ mRNA is consistent with inhibition of *XBP1* mRNA splicing (S7A Fig). We then used adenoviral forced expression of XBP1s to induce mRNAs encoding components of the SRP-dependent proximal ER in human islets. Significant expression of GFP was observed at 5 d after infection with *Ad-GFP*, indicating efficient Ad-mediated expression in human islets (S7B Fig). Interestingly, expression of *Ad-Xbp1s*, as opposed to its chemical inhibition, had the inverse effect on *INS* mRNA. Increased XBP1s was sufficient to increase *INS* mRNA levels, presumably because of increased capacity for mRNA recruitment to the ER and cotranslational translocation (S7A–S7C Fig). We then analyzed the effect of adenoviral expression of IRE1α, its mutants, and XBP1s on proinsulin production in human islets at the protein level. Expression of IRE1α mutants devoid of RNase function (IRE1α- ΔR or ΔCT) greatly reduced proinsulin levels compared to controls (S7D Fig). The results support the conclusion that positive regulation of proinsulin production by IRE1α is conserved between mice and humans.

## Discussion

Taken together, these results demonstrate glucose-stimulated IRE1α splicing of *Xbp1* mRNA in β cells induces expression of mRNAs encoding proteins important for proinsulin biosynthesis and many other intracellular processes essential for insulin biogenesis that include but are not limited to ribosome recruitment to the ER, cotranslational translocation, and signal peptide cleavage. Inversely, the IRE1α/XBP1s pathway is required to protect the β cell from expression of mRNAs encoding functions related to ER stress, oxidative stress, and inflammation. These results also show that the IRE1α- and glucose-dependent changes in mRNA abundance are important for a myriad of intracellular processes beyond the GO analysis enriched group of mRNAs responsible for expansion of the proximal ER. However, we focused on the most significant functional defect because it is the rate-limiting step in proinsulin synthesis upon glucose stimulation of the β cell (S8A and S8B Fig).

Although the UPR was originally characterized as an adaptive response to protein misfolding in the ER [55–57], to the best of our knowledge to date, there is no evidence that supports that physiological stimuli, such as glucose, cause transcriptional remodeling by IRE1α-mediated splicing of *Xbp1* mRNA to increase the demand for ER protein-folding capacity. Our results support the notion that glucose stimulation of β cells requires IRE1α mediated splicing of *Xbp1* mRNA to increase entry into and expansion of the SRPR/SSR-dependent secretory pathway’s capacity to accommodate increased preproinsulin synthesis, processing to proinsulin, folding, trafficking, and secretion (S6A Fig). Specifically, in the absence of IRE1α, there was defective glucose-stimulated induction of 12 mRNAs encoding functions in cotranslational translocation, ribosome subunits, and ribosome recruitment to the ER, proinsulin synthesis, and preproinsulin signal peptide cleavage. Because these processes represent the rate-limiting steps in cotranslational translocation and since multiple mRNAs in this functional group were significantly altered, we postulate that in aggregate the reduced expression of these mRNAs combine to cause the majority of the diabetic phenotypes we report for the *KO* mice. Specifically, we demonstrated at the functional levels that IRE1α is required for four processes critical for insulin biogenesis in mature β cells: (1) proinsulin mRNA translation, (2) ribosome recruitment and structure, (3) signal peptide cleavage, and (4) suppression of oxidative/inflammatory stress causing mRNAs.

Intriguingly, β cells are more sensitive to loss of *Ire1α* than fibroblasts or hepatocytes [20,39,40], possibly because of the rigors of daily periodic postprandial increases in preproinsulin synthesis coupled with basally low levels of antioxidant enzymes [58]. Because mRNA translation is compromised in the *KO* islets, the source of ROS is likely not uncontrolled protein synthesis, as was previously shown to occur upon elimination of eIF2α phosphorylation in the β cell [59]. In contrast, the *Ire1α-KO* islets contained higher mRNA levels for oxido-reductases that produce ROS, such as the LOXs, PDIAs, and NOS2, and that may contribute to the increased ROS within the null islets. *Ire1α*-deletion also increased TNC mRNA and protein levels. Alternatively, the loss of IRE1α-dependent antioxidant enzymes, such as selenoprotein S (SELS), may increase ROS in the *KO* islets. Regardless of the source of ROS or lack of protection, glucose tolerance was restored by feeding the *KO* mice a diet supplemented with the antioxidant BHA, indicating that ROS contribute to the β cell failure upon *Ire1α* deletion.

Previously, it was presumed that tripartite UPR signaling coordinates adaptation through regulation of protein synthesis and gene expression [60]. However, it is becoming evident that each UPR sensor has evolved to fulfill specific requirements in unique cell types. In the β cell, IRE1α-mediated *Xbp1* mRNA splicing and PERK-mediated eIF2α phosphorylation [9] are essential to maintain the structural integrity of the ER, preserve glucose responsiveness, and prevent oxidative damage, whereas ATF6α is dispensable [26,28,61]. Although both IRE1α and PERK are required to expand β cell mass through neonatal development [9,25,30], there are significant differences in mature β cells that are void of PERK/eIF2α-P versus IRE1α/XBP1s. *Perk* deletion or Ser51Ala mutation in eIF2α causes uncontrolled protein synthesis and decreases expression of the β cell-specific *Pdx1*, *MafA*, and *Ins1/2* mRNAs [9,25]. In contrast, *Ire1α* deletion reduces expression of mRNAs encoding proteins involved in ribosome recruitment to the ER, mRNA translation, translocation, and signal peptide cleavage without reducing expression of β cell-specific mRNAs. However, disruption of either PERK or IRE1α signaling in the β cell disrupts ER protein folding and trafficking to deplete insulin granules and cause oxidative stress and β cell failure. Whereas the PERK/eIF2α pathway is an important brake for the β cell secretory pathway, the IRE1α/XBP1s is a critical accelerator for increasing proinsulin synthesis in response to higher blood glucose. We propose that IRE1α/XBP1s evolved to expand the capacity for specialized secretory cells.

In summary, these findings demonstrate IRE1α/XBP1s is required for β cell function and should be considered in light of new therapeutic approaches that rely on IRE1α inhibition because IRE1α-dependent splicing of *Xbp1* mRNA is the only known conserved IRE1α RNase activity to all mammalian cell types [62–65].

## Materials and Methods

### Ethics Statement

Sanford Burnham Prebys Medical Discovery Institute (SBPMDI) follows the “Guide for the Care and Use of Laboratory Animals: Eighth Edition” standards. The Institute’s Animal Care & Use Program is accredited by AAALAC International, and a Multiple Project Assurance A3053-1 is on file in the OLAW, DHHS. Euthanasia is consistent with the recommendations of the 2013 AVMA Guidelines for the Euthanasia of Animals. All animal care and procedures were conducted according to the protocols and guidelines approved by the University of Michigan Committee on the Use and Care of Animals (UCUCA) and the SBPMDI, as well as by the Institutional Animal Care and Use Committee (IACUC) divisions of the American Association for Laboratory Animal Science (AALAS). IOur AAALAC number is 000710, and our mouse protocol number is 14–036. The human islets were sourced from the Clinical Islet Laboratory at the University of Alberta/Alberta Health Sciences Sanford Burnham Prebys Medical Discovery Institute IRB Code: 100894XX. Islet donors’ written consent was given to Clinical Islet Laboratory at the University of Alberta/Alberta Health Sciences.

### Mouse Husbandry

All animal care and procedures were conducted according to the protocols and guidelines approved by the UCUCA and the SBPMDI, as well as by the IACUC division of the AALAS.

### qRT-PCR

Mouse and human qRT-PCR primer sequences specific to total levels of mRNAs were obtained from the Harvard Primer Bank: http://pga.mgh.harvard.edu/primerbank/. See online supporting methods for additional details. The primers used to detect the presence and absence of the *Ire1α* floxed allele are as follows: FWD-cctacaagagtatgtggagc, REV-ggtctctgtgaacaatgttgagag. Spliced-specific *Xbp1* primers are as follows: FWD-gagtccgcagcaggtg, REV-gtgtcagagtccatggga. Unspliced-specific *Xbp1* primers are as follows: FWD-ctcagactatgtgcacctct (within the 26 nt intron), REV- catgactgggtccaagttgtccag.

### Conventional PCR Primers Flanking the 26 nt Intron in XBP1 mRNA

The primer sequences for XBP1 flanking PCR are FWD-ccttgtggttgagaaccagg, REV-gtgtcagagtccatggga amplicon + 211 bp (unspliced) and 185 bp (spliced).

### Blood Glucose Measurements and Pancreatic Proinsulin and Insulin Content

Mice were fasted for 4 h, and then fasted blood glucose measurements were recorded. Glucose tolerance tests (GTTs) were performed as previously described by intraperitoneally injecting a glucose solution of 2 g/kg by body weight and recording tail-vein blood measurements over time using a digital glucometer [9]. Insulin and proinsulin content was determined by diluting acid ethanol extracts from the pancreas at 50 mg/ml 1:200 in sample resuspension buffer provided by the ALPCO ELISA kits for insulin (Cat. 80-INSHU-E01.1) and proinsulin (Cat. 80-PINMS-E01)

### EM

Freshly dissected pancreas was fixed in Sorenson’s buffer and processed by the University of Michigan Electron Microscopy Core facility. Blind scoring of insulin granules was performed using Cell Profiler software.

### Adenoviral Infections

Experiments utilizing adenoviruses were performed in triplicate with graphs representing the average of all experiments except the human islets where the individual’s age is stated within the legends. Mouse islets were infected with adenovirus at 24 h post-isolation, and human islets were typically received 3 d postmortem and after one night in media were infected with adenoviruses. For infection of islets and the immortalized *Ire1α* ^*Fe/Fe*^ β cell line, 50 and ten plaque forming units per cell were used, respectively. At 72 h postadenoviral infection, analysis by pulse-chase and measurement for oxidative stress and HODEs was performed as described [9].

### mRNA-Seq Transcriptome Sequencing

The Illumina Genome Analyzer II was utilized to analyze 200 nt long, fragmented mRNA converted to cDNA (50 ng/individual) purified from islets of five individual mice per genotype according to Illumina mRNA-Seq kit (Part# 1004898). The islet mRNA-Seq data has been deposited to the SRA Study Accession: http://www.ncbi.nlm.nih.gov/sra/?term=SRP041246, and the bioproject website is http://www.ncbi.nlm.nih.gov/bioproject/242958.

### Polysome Profiling

Polysome profiles were obtained byt reating cells with 0.1 mg/mL cycloheximide (CHX) for 10 min at 37°C, washed twice with ice-cold PBS-CHX (phosphate buffered saline containing 0.1 mg/mL CHX), and harvested using polysome lysis buffer (20 mM Tris-HCl pH 7.5, 100 mM NaCl, 10 mM MgCl_2_, 0.4% IGEPAL, 50 μg/mL CHX, protease inhibitors, and RNaseIn). Lysates were clarified by centrifugation at 13,000 × *g* for 10 min at 4°C. Equal amounts of clarified lysates based on the absorption at 260 nm were layered onto 10%–50% sucrose gradient (prepared in 20 mM Tris-HCl pH 7.5, 100 mM NaCl, 10 mM MgCl_2_, 50 μg/mL CHX) and centrifuged in an SW41-Ti rotor at 40,000 rpm for 2 h at 4°C. Fractions were collected using a Bio-Rad fraction collector, and the amount of total RNA in each fraction was measured using a NanoDrop spectrophotometer [66].

### Subcellular Fractionation

The Pierce kit catalog #78840 was used to isolate subcellular fractions.

## Supporting Information

S1 Data Raw data to all quantitative experiments. 14 sheets/experiments.
(XLSX)

S2 Data Mass spectrometry of islets expressing dominant negative IRE1α-K907A versus β-Gal control adenovirus. (XLSX)

S3 Data mRNA-Seq expression and GO data for the *Ire1α*- and glucose-dependent mRNAs. (XLSX)

S4 Data mRNAs overlapping between our islet mRNA-Seq, XBP1 chromatin immunoprecipitation sequencing (ChIP-Seq), and RIDD studies. (XLSX)

S1 Fig Tam-induced *Ire1α* deletion in adult β cells causes a diabetic phenotype. (A) GTTs at 2, 4, 6 (Fig 1B), 8, and 20 wk post-Tam injection. All data and statistics including a time course depiction of the areas under the GTT curves are provided within S1 Data. Results of the areas under the curve: ([*WT* ^*Fe/+*^, *Het-I* ^*Fe/+; Cre*^, *Het-B* ^*Fe/-*^, and *KO* ^*Fe/-; Cre*^], [*t*-test significance *p*-value]). GTTs ([2 wk, *n* = 0, 4, 4, 7], [*p* = 0.0035; *KO* ^*Fe/-; Cre*^ versus all controls]), ([4 wk, *n* = 10, 3, 9, 8], [*p* = 0.00021; *KO* ^*Fe/-; Cre*^ versus *WT*]), ([6 wk, *n* = 8, 3, 6, 8], [*p* = 0.00053; *KO* versus *WT*]), ([8 wk, *n* = 10, 3, 9, 8], [*p* = 0.000572; *KO* ^*Fe/-; Cre*^ versus *WT*]), ([20 wk, *n* = 12, 3, 8, 8], [*p* = 0.000124; *KO* ^*Fe/-; Cre*^ versus *WT*]). (B) ELISA for serum proinsulin from the samples analyzed for insulin in Fig 1C ([*n* = 7, 7], [p = 0.0364; *KO* ^*Fe/-; Cre*^ versus *Het-B*]), ([*n* = 7, 4], [*p* = 0.0641; *KO* ^*Fe/-; Cre*^ versus *WT*]) and ([*n* = 7,4], [*p* = 0.0450; *KO* ^*Fe/-; Cre*^ versus *Het-I*]). (C) Immunofluorescence microscopy of *WT* and *KO* ^*Fe/-; Cre*^ islets for insulin (red), proinsulin (green), and DAPI (blue). Additional results also depicted in Fig 1D. Scale bar, 100 μm. The inset of the *KO’s* ^*Fe/-; Cre*^ merged panel has had the brightness increased 2-fold in order to better visualize the islet. *KO* ^*Fe/-; Cre*^ islets with partial proinsulin and insulin staining are shown below. (D) Serum dopamine levels measured by ELISA indicated no significant difference (*WT* ^*Fe/+*^; *n* = 5, *Het-B* ^*Fe/-*^; *n* = 7, *Het-I* ^*Fe/+; Cre*^; *n* = 5 and *KO* ^*Fe/-; Cre*^; *n* = 6). (E) Immunofluorescence microscopy of *WT* ^*Fe/+*^ and *KO* ^*Fe/-; Cre*^ arcuate nuclei of the hypothalamus (outlined in white) for growth hormone-releasing hormone (GHRH, red), Cre recombinase (Cre, green), and for nuclei (Hoechst, blue) of the hypothalamus. Cre was detected in the *KO* ^*Fe/-; Cre*^ brains; however, the GHRH signal was not significantly reduced.
(TIF)

S2 Fig *Ire1α* deletion causes ER stress in β cells. (A) EM at 2 wk post-Tam injection of whole islets (top), β cells (middle), and organelles (bottom). The lower right panel depicts insulin granule depletion in the *KO* ^*Fe/-; Cre*^ as measured using Cell Profiler quantification ([*p* = 0.0002] [*Het-I* ^*Fe/+; Cre*^; *n* = 10, *KO* ^*Fe/-; Cre*^; *n* = 14]) (bottom, right). Pyknotic nuclei are indicated by yellow arrows in the *KO* ^*Fe/-; Cre*^ micrograph’s middle panel. Lamellar, autophagic-like structures and distended mitochondria are shown in the bottom panel. Scale bars, (top; 700x = 10 μm), (middle; 10,500x = 2 μm) and (bottom; 25,000x–75,000x; top row = 1.0 μm, all other scale bars = 0.5 μm).
(TIF)

S3 Fig *Ire1α* deletion causes ER stress in β cells (continued). (A) Immunofluorescence costaining of MAFA (red), proinsulin (green), insulin (blue), and PDX1 (orange) in *Het-I* ^*Fe/+; Cre*^ versus *KO* ^*Fe/-; Cre*^ islets at 6 wk post-Tam injection. Reduced total MAFA signal leads to reduced nuclear MAFA despite increased mRNA expression in *KO* ^*Fe/-; Cre*^ islets (Figs 1J and 3A and S4A Fig), whereas PDX1 nuclear localization is unaffected. Pink nuclei in the DAPI merged panels (third from right) represent MAFA plus DAPI double-positive nuclei that were present only in the *Het-I* ^*Fe/+; Cre*^, whereas in the last two panels PDX1 levels and nuclear localization were not significantly reduced in the *KO* ^*Fe/-; Cre*^ (white arrows). Scale bar, 20 μm at 200x magnification. (B) Immunofluorescence costaining of KDEL and GLUT2 in *WT* ^*Fe/+*^, *Het-I* ^*Fe/+; Cre*^, and *KO* ^*Fe/-; Cre*^ islets. An additional example is shown in Fig 2B. Scale bars, (top; 400x = 50 μm), (middle; 1,000x = 10 μm), (lower left; 3,500x = 2 μm), and (lower right; 8,200x = 1 μm). Increased yellow signal at the interface between GLUT2-red and KDEL-green was apparent in the *KO* ^*Fe/-; Cre*^ islets. Red blood cells (RBCs) are indicated by blue arrows in the 1000x, middle panel.
(TIF)

S4 Fig mRNA sequencing identifies IRE1α/XBP1s- and glucose-dependent mRNAs in islets. (A) qRT-PCR analysis of islet-specific and ER-stress mRNAs to validate mRNA-Seq data. Error bars represent average deviation of the technical replicates for the cDNA pooled from the islets of five littermate male mice (*n* = 5) at 6 wk post-Tam. (B) Overlapping genes from the islet mRNA-Seq study and a previous ChIP-Seq study performed on XBP1. (C) Overlapping mRNAs from the *KO* islet mRNA-Seq study and a “RIDD” study that examined the three cell lines shown. First, the overlap between the mRNAs identified in the RIDD study was determined (left Venn). Next, a Venn diagram was generated to identify overlap between the combined RIDD targets and mRNAs reduced or increased by *Ire1α* deletion during high glucose (middle Venn). The mRNAs shared between studies and unique to islet mRNA-Seq are listed on the right. The 1,346 newly identified mRNAs exhibiting the “RIDD” trend in islets were analyzed by the DAVID GO program and presented in S4 Data.
(TIF)

S5 Fig *Ire1α* deletion in β cells causes oxidative stress, inflammation and fibrosis. (A and B) mRNA-Seq expression values for mRNAs decreased in 18 mM glucose incubated *WT* ^*Fe/+*^ islets that were increased in *KO* ^*Fe/-; Cre*^ islets ([*n* = 5, 5, 5], [18 mM *KO* ^*Fe/-; Cre*^], [*p* = < 0.01]). The *Het-I* ^*Fe/+; Cre*^ mRNA-Seq expression data are presented in the supporting figures to demonstrate that the RIP-Cre allele is not responsible for the mRNAs we attribute to the absence of IRE1α in β cells. (A) Previously identified as RIDD targets (top panel). (B) mRNAs of the same trend in which glucose caused reduction in the *WT* ^*Fe/+*^ and accumulation in the *KO* ^*Fe/-; Cre*^ that are novel to islet mRNA-Seq. Additional mRNAs with this expression trend are depicted in Fig 4A. The GO terms associated with these mRNAs were enriched for ECM proteins, catabolic enzymes, and inflammation (Fig 3C [right] and 3D [right]). (C) mRNA-Seq expression values for oxidative stress response mRNAs (NOS2 and GPX2) and BIP exhibited glucose dependence that accumulated without functional IRE1α. Error bars represent the *p*-values from the cDNA of five mice per genotype. (D) Islets from 5 and 7-mo-old *WT* ^*Fe/Fe*^ and *KO* ^*Fe/Fe; Cre*^ mice at 15 wk post-Tam with or without BHA diet for 3 wk analyzed for anti-TNC. TNC reactivity was increased in the *KO* islets and was reduced to control levels by BHA diet. (*WT* ^*Fe/Fe*^ [*n* = 5 w/ BHA], [*n* = 4 regular chow], [*p* = 0.035]), (*KO* ^*Fe/Fe; Cre*^ [*n* = 5 with BHA], [*n* = 6 without BHA]) *p* = n.s. (E) Additional examples are shown in Fig 4D for Masson's trichrome collagen stain of islets showing increased blue collagen surrounding the *KO* ^*Fe/Fe; Cre*^ islet at 18 wk post-Tam injection (15 wk post-Tam, 3 wk with or without BHA diet) that was reduced by BHA diet.
(TIF)

S6 Fig IRE1α- and glucose-dependent mRNAs encode diverse intracellular functions other than the proximal ER. (A) mRNA-Seq values for UPR target mRNAs. All mRNA-Seq values are relative to the 1.0-fold change (Fc) of the 6 mM *WT* ^*Fe/+*^ sample. (B) mRNA-Seq values for newly identified *Ire1α*- and glucose-dependent mRNAs identified that do not cluster with the 12 proximal ER mRNAs. Additional examples are depicted in Fig 5A, upper panel. (C) Polysome profiles for the *Ire1α* ^*Fe/Fe*^ insulinoma line. The results demonstrate *Ire1α* deletion reduces the polysome/monosome ratio with an overall increase in total ribosomes that was also observed by western blotting (Fig 5E and S6D Fig). Polysome profiles were prepared by ultracentrifugation of lysates over sucrose gradients (*n* = 2). (D) RPS9 western blots for the 12 mM samples from the experiment in Fig 5E (*n* = 3). (E) The defective recruitment of RPS9 protein to the membranous fraction upon high glucose in the absence of IRE1*α* could not be explained at the mRNA level within islets.
(TIF)

S7 Fig IRE1α/XBP1s is necessary for glucose-stimulated insulin mRNA induction in human islets. (A) qRT-PCR on human islets after 24 h incubation in 6 mM versus 18 mM glucose containing media with increasing amounts (1 μM, 3.3 μM, and 10 μM) of IRE1α inhibitor (MNKD8866). Results are representative of islets from three male cadavers at 17, 45, and 21 y of age and one female at age 52 (*n* = 4). The results from the 17-y-old male are presented because they were the most viable and receptive to glucose stimulation. (B) Islets from 17-y-old male at 5 d post-infection with 50 plaque-forming units of *Ad-GFP*. The results indicate expression occurs within the islet core. (C) qRT-PCR on human islets for mRNAs encoding ER proximal components identified by murine islet mRNA-Seq to be *Ire1α* and glucose dependent. Human islets were infected for 5 d as in S7B before RNA isolation. Results represent the islets from three male cadavers at 17, 45, and 21 y of age and one female at age 52 (*n* = 4). (D) Western blotting of human islet lysates for the ER-associated degradation member osteosarcoma 9 (OS9), GRP94 and BIP (KDEL-proteins), proinsulin, and tubulin. OS9 was detected by mass spectrometry (Fig 3D) as decreased in IRE1α-deficient islets, whereas it was induced by *Ad-Xbp1s* in human islets (S7D Fig). Isolated islets were infected with adenoviruses encoding IRE1α, IRE1α mutants (kinase-K599A, RNase-K907A/ΔR, and C-terminus truncation-ΔCT), XBP1s, ATF6α, and GFP and were compared to the noninfected control after 5 d by western blot. Results are representative of islets from two male cadavers at 17 and 21 y of age and one female at age 52 (*n* = 3). The results from the 17-y-old male are shown.
(TIF)

S8 Fig Glucose stimulation of β cells requires the IRE1α/XBP1s to increase insulin production. (A) Depiction of the events that occur upon glucose stimulation of β cells in the presence (top) and absence (bottom) of IRE1α. (B) Summary of the mRNAs pathways found to be IRE1α and glucose dependent.
(TIF)

S1 Text Supporting Materials and Methods. Details are provided for oxidized lipid, islet steady-state isotopic labeling, mass spectrometry analysis, Masson’s trichrome stain, cDNA synthesis for qRT-PCR, GTTs, and insulin and proinsulin measurements, immunofluorescence and immunohistochemical microscopy, antibodies used, proinsulin synthesis, bioinformatics analysis, and GO.
(DOC)

## Abbreviations

AALAS: American Association for Laboratory Animal Science
ATF6α: activating transcription factor 6 alpha
BHA: butylated hydroxyanisole
BIP: immunoglobulin binding protein
bZIP: basic-leucine zipper-containing
cDNA: complementary DNA
ChIP-Seq: chromatin immunoprecipitation sequencing
CHX: cycloheximide
COL: collagen
CPE: carboxypeptidase E
DAVID: the Database for Annotation, Visualization and Integrated Discovery
DIDMOAD: diabetes insipidus, diabetes mellitus, optic atrophy, and deafness
ECM: extracellular matrix
EM: electron microscopy
ER: endoplasmic reticulum
ERAD: ER-associated protein degradation
GHRH: growth hormone-releasing hormone
GO: gene ontology
GPX: glutathione peroxidase
GSIS: glucose-stimulated insulin secretion
GTT: glucose tolerance test
HODE: hydroxyl-octadecadienoic acid
IACUC: Institutional Animal Care & Use Committee
IHC: immunohistochemistry
iNOS: inducible nitric oxide synthase
INS1 and INS2: insulin
IP: immunoprecipitation
IRE1α: inositol-requiring enzyme 1 alpha
LOX: lysyl-oxidase
MAFA: v-maf avian musculoaponeurotic fibrosarcoma oncogene homolog A
miRNA: microRNA
mRNA-Seq: mRNA sequencing
OS9: osteosarcoma 9
PC1 and PC2: prohormone convertases
PDIA4 and PDIA5: protein disulfide isomerases 4 and 5
PERK: PKR-like ER kinase
qRT-PCR: quantitative real-time PCR
RBC: red blood cell
RIDD: regulated IRE1α-dependent degradation
RNase: ribonuclease
ROS: reactive oxygen species
SBPMDI: Sanford Burnham Prebys Medical Discovery Institute
SELS: selenoprotein S
SRP: signal recognition particle
SSR: signal sequence receptor
T2D: type 2 diabetes
Tam: Tamoxifen
TF: transcription factor
TNC: tenascin C
TUNEL: terminal deoxynucleotidyl transferase dUTP nick end labeling
UCUCA: University of Michigan Committee on Use and Care of Animals
UPR: unfolded protein response
WFS1: Wolfram syndrome protein 1
XBP1: X-box-binding protein 1
