# Supplementary figures and images for: The IRE1α/XBP1s Pathway Is Essential for the Glucose Response and Protection of β Cells
Source: PLoS Biol. 2015 Oct 15;13(10):e1002277. doi: 10.1371/journal.pbio.1002277 (PMC4607427; doi:10.1371/journal.pbio.1002277)

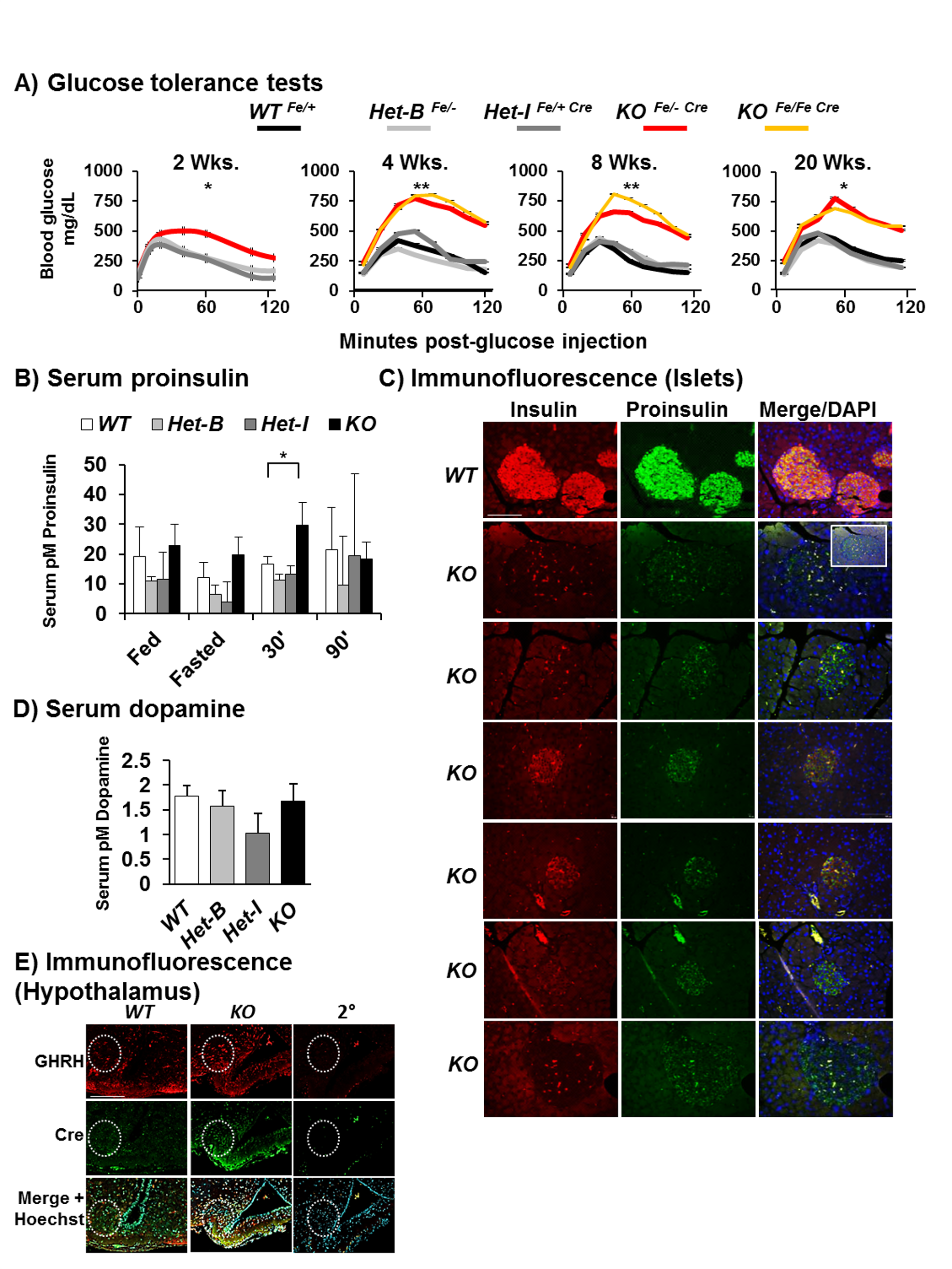

Supplement: S1 Fig — (A) GTTs at 2, 4, 6 (Fig 1B), 8, and 20 wk post-Tam injection. All data and statistics including a time course depiction of the areas under the GTT curves are provided within S1 Data. Results of the areas under the curve: ([WT Fe/+, Het-I Fe/+; Cre, Het-B Fe/-, and KO Fe/-; Cre], [t-test significance p-value]). GTTs ([2 wk, n = 0, 4, 4, 7], [p = 0.0035; KO Fe/-; Cre versus all controls]), ([4 wk, n = 10, 3, 9, 8], [p = 0.00021; KO Fe/-; Cre versus WT]), ([6 wk, n = 8, 3, 6, 8], [p = 0.00053; KO versus WT]), ([8 wk, n = 10, 3, 9, 8], [p = 0.000572; KO Fe/-; Cre versus WT]), ([20 wk, n = 12, 3, 8, 8], [p = 0.000124; KO Fe/-; Cre versus WT]). (B) ELISA for serum proinsulin from the samples analyzed for insulin in Fig 1C ([n = 7, 7], [p = 0.0364; KO Fe/-; Cre versus Het-B]), ([n = 7, 4], [p = 0.0641; KO Fe/-; Cre versus WT]) and ([n = 7,4], [p = 0.0450; KO Fe/-; Cre versus Het-I]). (C) Immunofluorescence microscopy of WT and KO Fe/-; Cre islets for insulin (red), proinsulin (green), and DAPI (blue). Additional results also depicted in Fig 1D. Scale bar, 100 μm. The inset of the KO’s Fe/-; Cre merged panel has had the brightness increased 2-fold in order to better visualize the islet. KO Fe/-; Cre islets with partial proinsulin and insulin staining are shown below. (D) Serum dopamine levels measured by ELISA indicated no significant difference (WT Fe/+; n = 5, Het-B Fe/-; n = 7, Het-I Fe/+; Cre; n = 5 and KO Fe/-; Cre; n = 6). (E) Immunofluorescence microscopy of WT Fe/+ and KO Fe/-; Cre arcuate nuclei of the hypothalamus (outlined in white) for growth hormone-releasing hormone (GHRH, red), Cre recombinase (Cre, green), and for nuclei (Hoechst, blue) of the hypothalamus. Cre was detected in the KO Fe/-; Cre brains; however, the GHRH signal was not significantly reduced. (TIF) [file pbio.1002277.s005.tif]

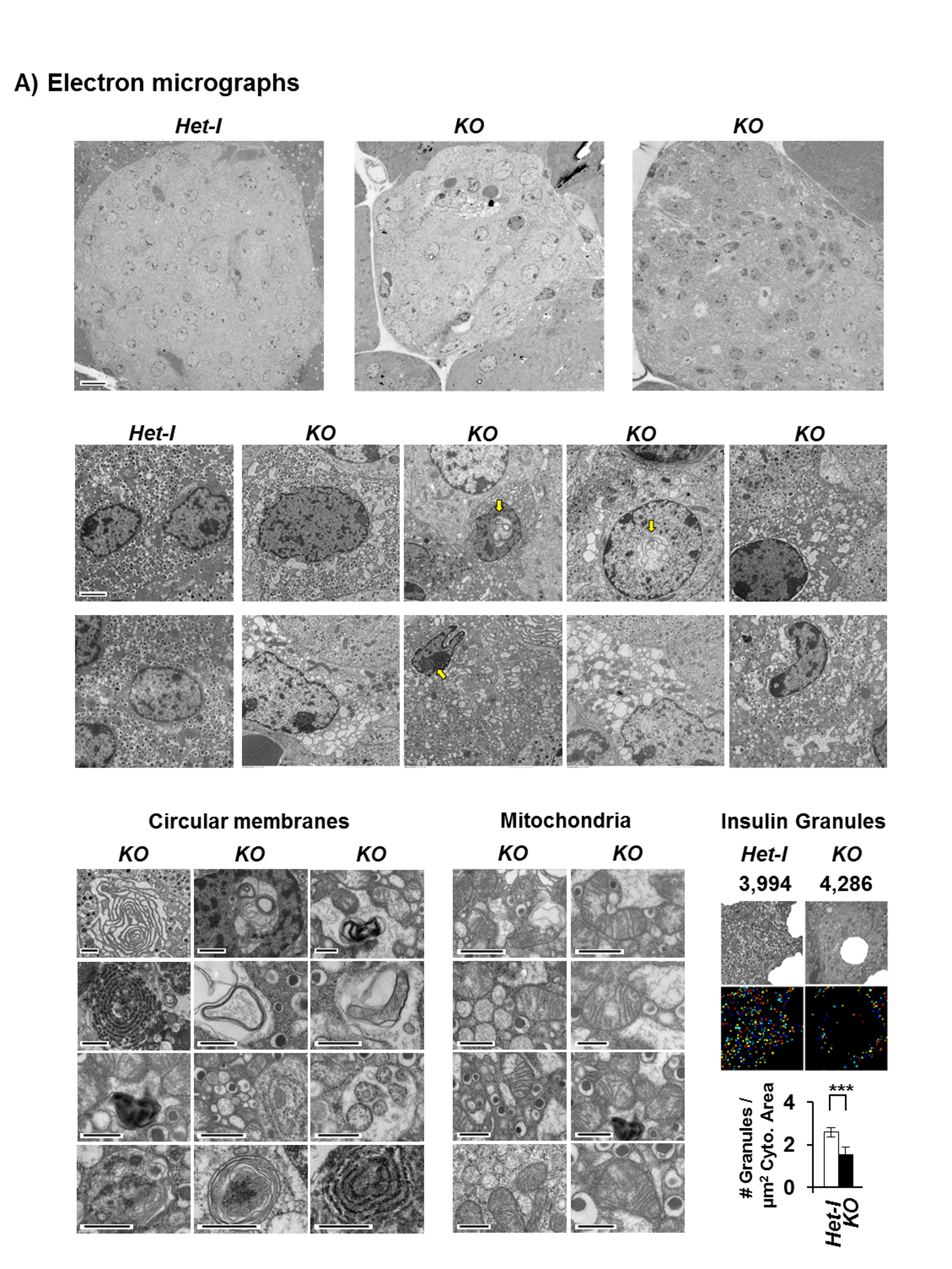

Supplement: S2 Fig — (A) EM at 2 wk post-Tam injection of whole islets (top), β cells (middle), and organelles (bottom). The lower right panel depicts insulin granule depletion in the KO Fe/-; Cre as measured using Cell Profiler quantification ([p = 0.0002] [Het-I Fe/+; Cre; n = 10, KO Fe/-; Cre; n = 14]) (bottom, right). Pyknotic nuclei are indicated by yellow arrows in the KO Fe/-; Cre micrograph’s middle panel. Lamellar, autophagic-like structures and distended mitochondria are shown in the bottom panel. Scale bars, (top; 700x = 10 μm), (middle; 10,500x = 2 μm) and (bottom; 25,000x–75,000x; top row = 1.0 μm, all other scale bars = 0.5 μm). (TIF) [file pbio.1002277.s006.tif]

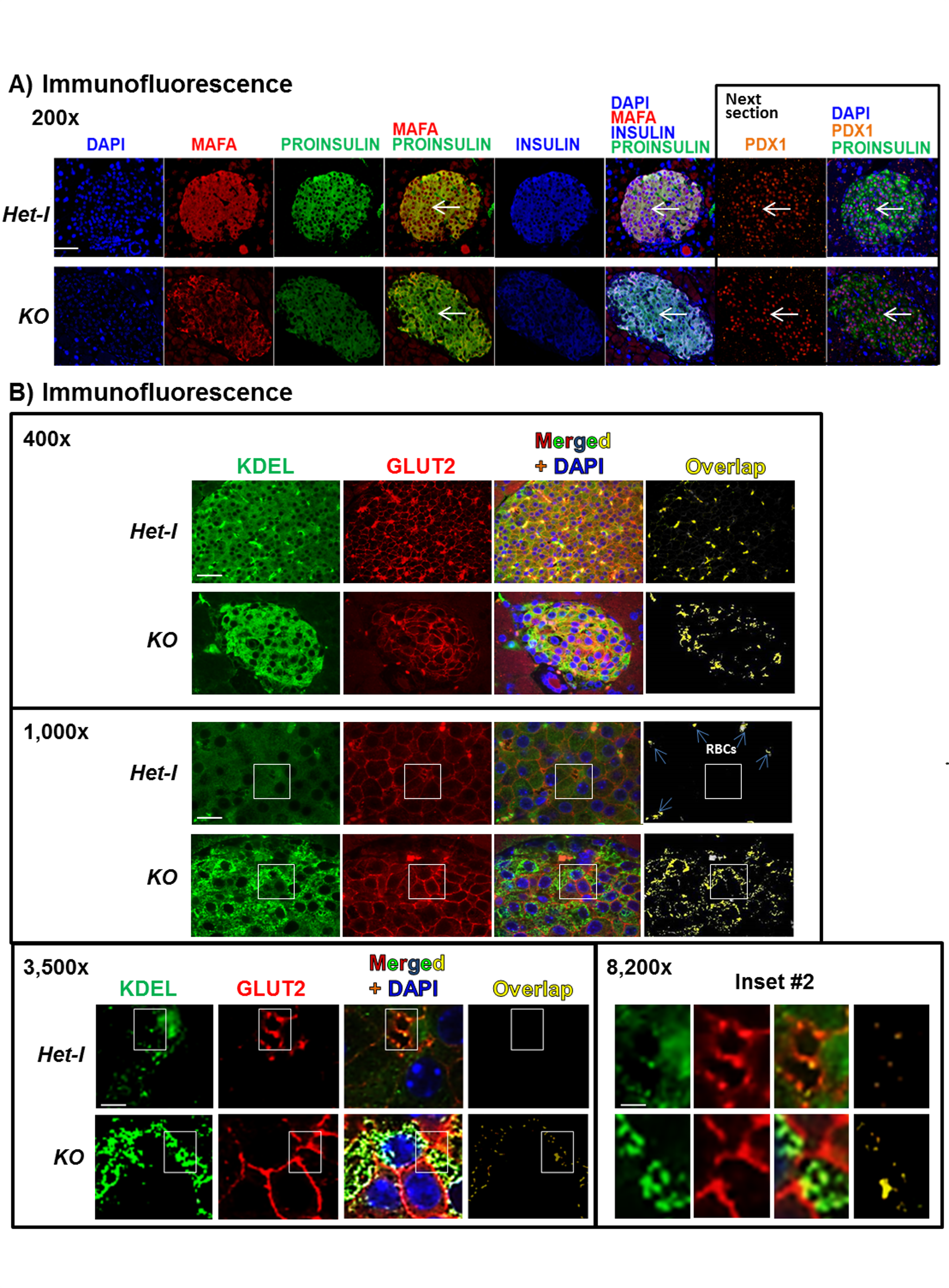

Supplement: S3 Fig — (A) Immunofluorescence costaining of MAFA (red), proinsulin (green), insulin (blue), and PDX1 (orange) in Het-I Fe/+; Cre versus KO Fe/-; Cre islets at 6 wk post-Tam injection. Reduced total MAFA signal leads to reduced nuclear MAFA despite increased mRNA expression in KO Fe/-; Cre islets (Figs 1J and 3A and S4A Fig), whereas PDX1 nuclear localization is unaffected. Pink nuclei in the DAPI merged panels (third from right) represent MAFA plus DAPI double-positive nuclei that were present only in the Het-I Fe/+; Cre, whereas in the last two panels PDX1 levels and nuclear localization were not significantly reduced in the KO Fe/-; Cre (white arrows). Scale bar, 20 μm at 200x magnification. (B) Immunofluorescence costaining of KDEL and GLUT2 in WT Fe/+, Het-I Fe/+; Cre, and KO Fe/-; Cre islets. An additional example is shown in Fig 2B. Scale bars, (top; 400x = 50 μm), (middle; 1,000x = 10 μm), (lower left; 3,500x = 2 μm), and (lower right; 8,200x = 1 μm). Increased yellow signal at the interface between GLUT2-red and KDEL-green was apparent in the KO Fe/-; Cre islets. Red blood cells (RBCs) are indicated by blue arrows in the 1000x, middle panel. (TIF) [file pbio.1002277.s007.tif]

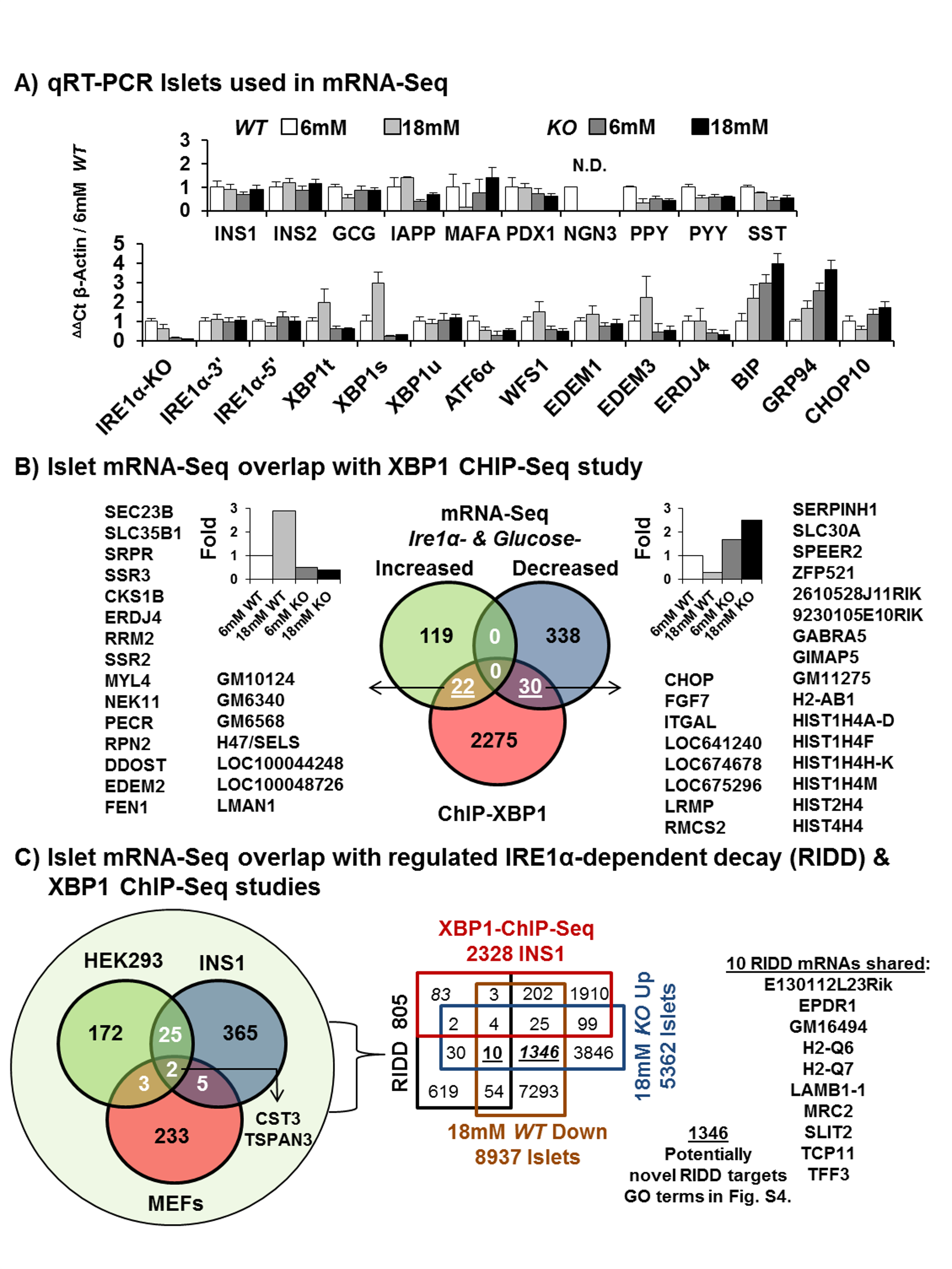

Supplement: S4 Fig — (A) qRT-PCR analysis of islet-specific and ER-stress mRNAs to validate mRNA-Seq data. Error bars represent average deviation of the technical replicates for the cDNA pooled from the islets of five littermate male mice (n = 5) at 6 wk post-Tam. (B) Overlapping genes from the islet mRNA-Seq study and a previous ChIP-Seq study performed on XBP1. (C) Overlapping mRNAs from the KO islet mRNA-Seq study and a “RIDD” study that examined the three cell lines shown. First, the overlap between the mRNAs identified in the RIDD study was determined (left Venn). Next, a Venn diagram was generated to identify overlap between the combined RIDD targets and mRNAs reduced or increased by Ire1α deletion during high glucose (middle Venn). The mRNAs shared between studies and unique to islet mRNA-Seq are listed on the right. The 1,346 newly identified mRNAs exhibiting the “RIDD” trend in islets were analyzed by the DAVID GO program and presented in S4 Data. (TIF) [file pbio.1002277.s008.tif]

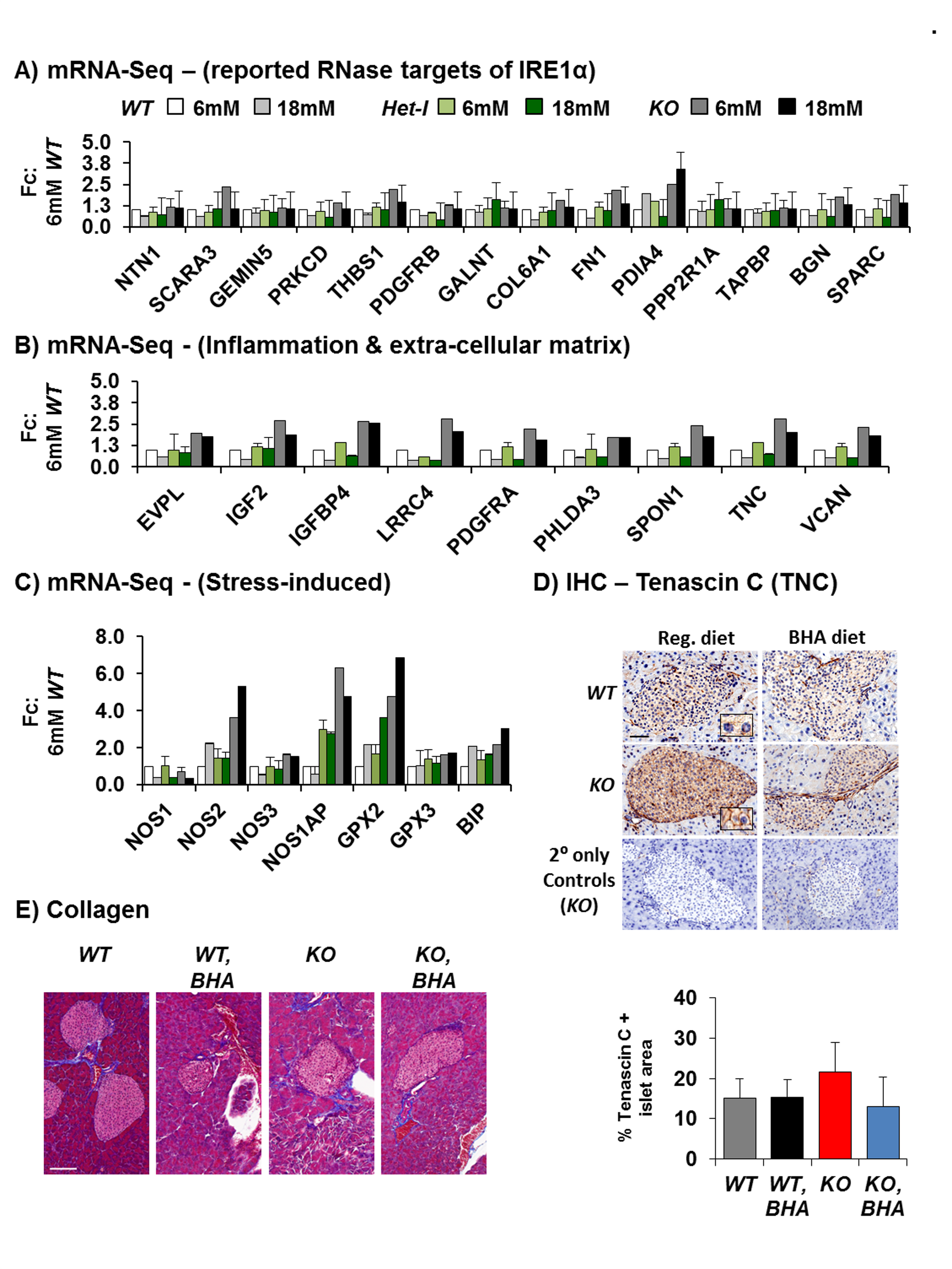

Supplement: S5 Fig — (A and B) mRNA-Seq expression values for mRNAs decreased in 18 mM glucose incubated WT Fe/+ islets that were increased in KO Fe/-; Cre islets ([n = 5, 5, 5], [18 mM KO Fe/-; Cre], [p = < 0.01]). The Het-I Fe/+; Cre mRNA-Seq expression data are presented in the supporting figures to demonstrate that the RIP-Cre allele is not responsible for the mRNAs we attribute to the absence of IRE1α in β cells. (A) Previously identified as RIDD targets (top panel). (B) mRNAs of the same trend in which glucose caused reduction in the WT Fe/+ and accumulation in the KO Fe/-; Cre that are novel to islet mRNA-Seq. Additional mRNAs with this expression trend are depicted in Fig 4A. The GO terms associated with these mRNAs were enriched for ECM proteins, catabolic enzymes, and inflammation (Fig 3C [right] and 3D [right]). (C) mRNA-Seq expression values for oxidative stress response mRNAs (NOS2 and GPX2) and BIP exhibited glucose dependence that accumulated without functional IRE1α. Error bars represent the p-values from the cDNA of five mice per genotype. (D) Islets from 5 and 7-mo-old WT Fe/Fe and KO Fe/Fe; Cre mice at 15 wk post-Tam with or without BHA diet for 3 wk analyzed for anti-TNC. TNC reactivity was increased in the KO islets and was reduced to control levels by BHA diet. (WT Fe/Fe [n = 5 w/ BHA], [n = 4 regular chow], [p = 0.035]), (KO Fe/Fe; Cre [n = 5 with BHA], [n = 6 without BHA]) p = n.s. (E) Additional examples are shown in Fig 4D for Masson's trichrome collagen stain of islets showing increased blue collagen surrounding the KO Fe/Fe; Cre islet at 18 wk post-Tam injection (15 wk post-Tam, 3 wk with or without BHA diet) that was reduced by BHA diet. (TIF) [file pbio.1002277.s009.tif]

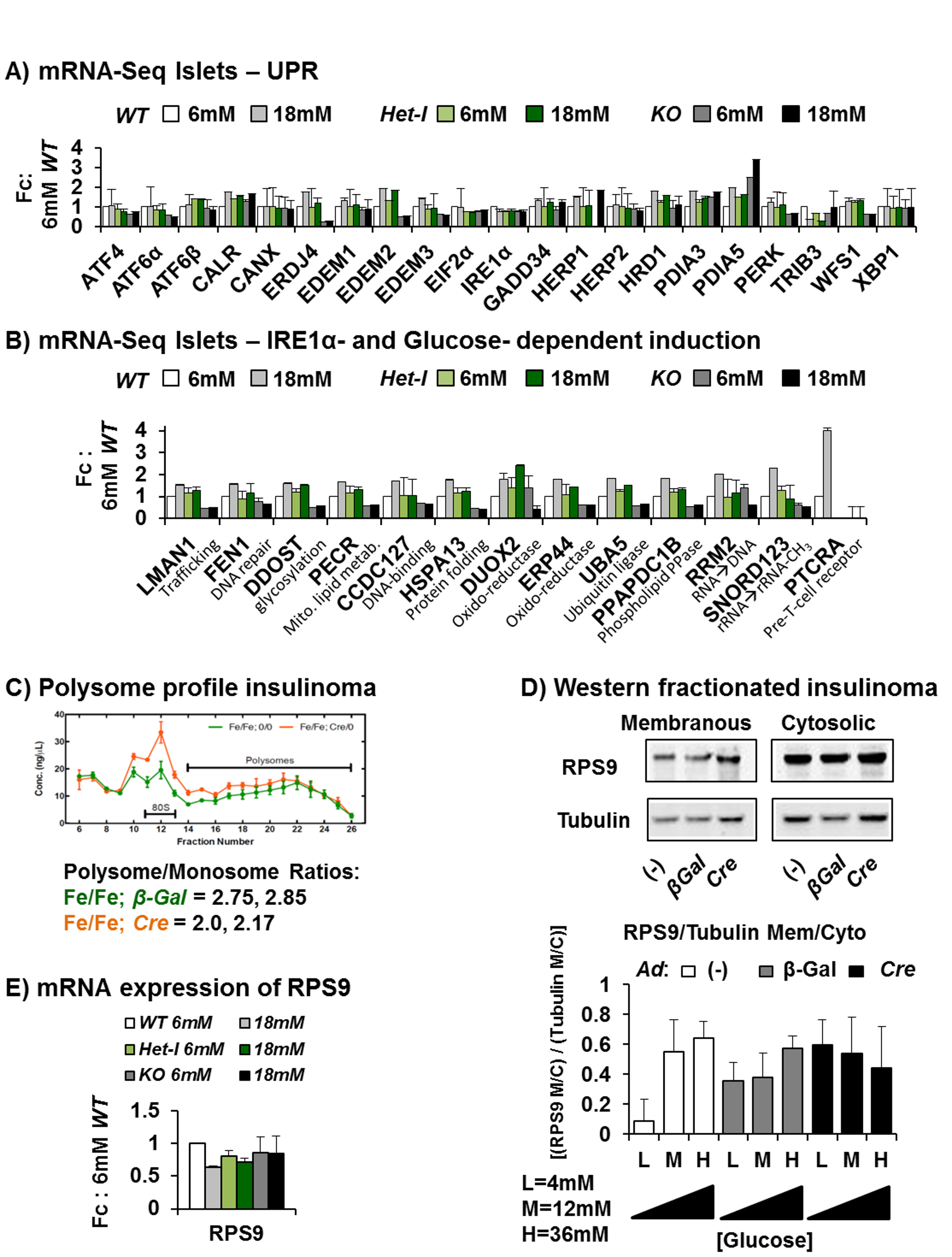

Supplement: S6 Fig — (A) mRNA-Seq values for UPR target mRNAs. All mRNA-Seq values are relative to the 1.0-fold change (Fc) of the 6 mM WT Fe/+ sample. (B) mRNA-Seq values for newly identified Ire1α- and glucose-dependent mRNAs identified that do not cluster with the 12 proximal ER mRNAs. Additional examples are depicted in Fig 5A, upper panel. (C) Polysome profiles for the Ire1α Fe/Fe insulinoma line. The results demonstrate Ire1α deletion reduces the polysome/monosome ratio with an overall increase in total ribosomes that was also observed by western blotting (Fig 5E and S6D Fig). Polysome profiles were prepared by ultracentrifugation of lysates over sucrose gradients (n = 2). (D) RPS9 western blots for the 12 mM samples from the experiment in Fig 5E (n = 3). (E) The defective recruitment of RPS9 protein to the membranous fraction upon high glucose in the absence of IRE1α could not be explained at the mRNA level within islets. (TIF) [file pbio.1002277.s010.tif]

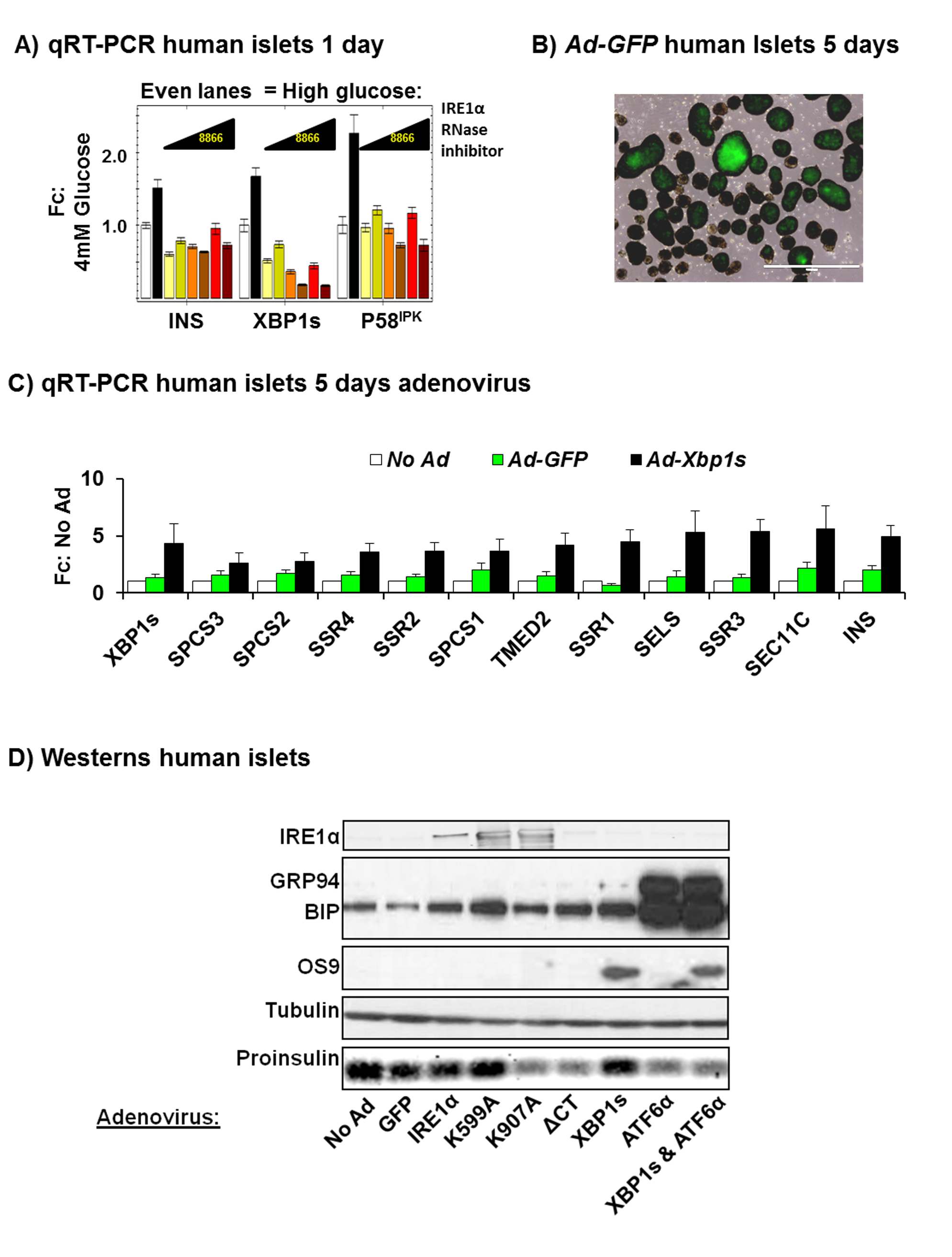

Supplement: S7 Fig — (A) qRT-PCR on human islets after 24 h incubation in 6 mM versus 18 mM glucose containing media with increasing amounts (1 μM, 3.3 μM, and 10 μM) of IRE1α inhibitor (MNKD8866). Results are representative of islets from three male cadavers at 17, 45, and 21 y of age and one female at age 52 (n = 4). The results from the 17-y-old male are presented because they were the most viable and receptive to glucose stimulation. (B) Islets from 17-y-old male at 5 d post-infection with 50 plaque-forming units of Ad-GFP. The results indicate expression occurs within the islet core. (C) qRT-PCR on human islets for mRNAs encoding ER proximal components identified by murine islet mRNA-Seq to be Ire1α and glucose dependent. Human islets were infected for 5 d as in S7B before RNA isolation. Results represent the islets from three male cadavers at 17, 45, and 21 y of age and one female at age 52 (n = 4). (D) Western blotting of human islet lysates for the ER-associated degradation member osteosarcoma 9 (OS9), GRP94 and BIP (KDEL-proteins), proinsulin, and tubulin. OS9 was detected by mass spectrometry (Fig 3D) as decreased in IRE1α-deficient islets, whereas it was induced by Ad-Xbp1s in human islets (S7D Fig). Isolated islets were infected with adenoviruses encoding IRE1α, IRE1α mutants (kinase-K599A, RNase-K907A/ΔR, and C-terminus truncation-ΔCT), XBP1s, ATF6α, and GFP and were compared to the noninfected control after 5 d by western blot. Results are representative of islets from two male cadavers at 17 and 21 y of age and one female at age 52 (n = 3). The results from the 17-y-old male are shown. (TIF) [file pbio.1002277.s011.tif]

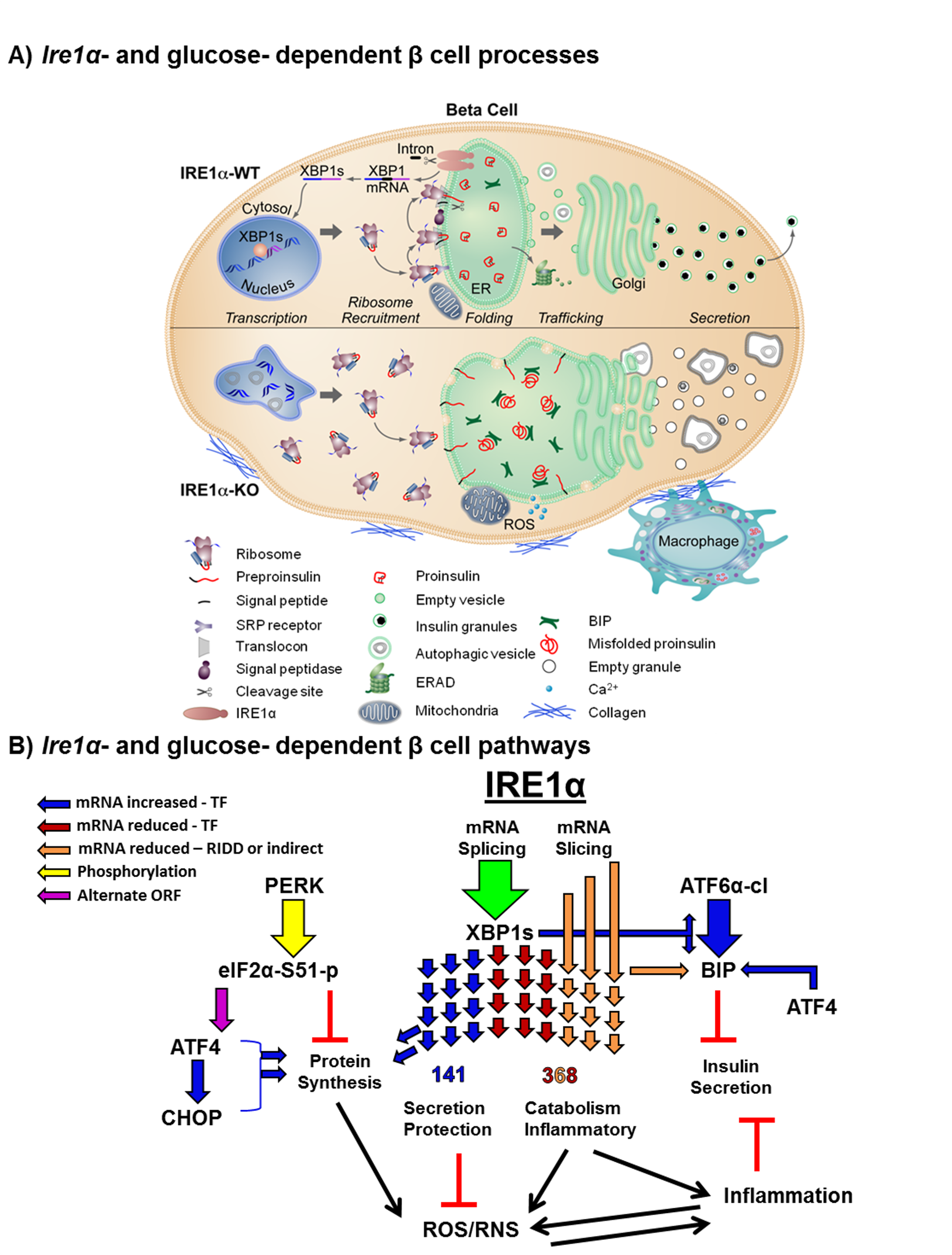

Supplement: S8 Fig — (A) Depiction of the events that occur upon glucose stimulation of β cells in the presence (top) and absence (bottom) of IRE1α. (B) Summary of the mRNAs pathways found to be IRE1α and glucose dependent. (TIF) [file pbio.1002277.s012.tif]
