# Supplementary material for: The IRE1α/XBP1s Pathway Is Essential for the Glucose Response and Protection of β Cells
Source: PLoS Biol. 2015 Oct 15;13(10):e1002277. doi: 10.1371/journal.pbio.1002277 (PMC4607427; doi:10.1371/journal.pbio.1002277)
Supplement: S1 Text — Details are provided for oxidized lipid, islet steady-state isotopic labeling, mass spectrometry analysis, Masson’s trichrome stain, cDNA synthesis for qRT-PCR, GTTs, and insulin and proinsulin measurements, immunofluorescence and immunohistochemical microscopy, antibodies used, proinsulin synthesis, bioinformatics analysis, and GO. (DOC) [file pbio.1002277.s013.doc]

**Supporting Materials and Methods**

- **HODE**
- Hydroxyoctadecadienoic acids (HODEs) were quantified by reverse-phase C-18 HPLC analysis of triphenylphosphine-reduced lipid extracts after base hydrolysis.
- **Islet steady-state isotopic labeling**
- Islets were incubated for 18 hours in 50µCi/ml [^35^S]-Cys/Met with 1/5 the normal amount of unlabeled Cys/Met containing RPMI media supplemented with 1% bovine serum albumin. Islets were washed with 700µl ice cold PBS twice and lysed in 2% SDS, 50mM Tris-Cl pH6.8, plus protease inhibitors. Denaturing SDS-PAGE sample loading was normalized by trichloracetate (TCA) precipitable counts. Filter paper TCA precipitation was used for scintillation counting of samples in duplicate.

**Mass spectrometry analysis**

Isolated islets infected with *Ad-β-Gal* and *Ad-∆R/K907A* for 72 hours then incubated with increased glucose (18mM) for 24 hours and twenty-five micrograms of 8M Urea protein extract was precipitated with 5x volume of cold acetone. The protein pellets were obtained by centrifuging at 14,000g for 10 min at 4 ℃, and then solubilized and reduced with 100mMTris-HCl/8M urea/5mM DTT. Cysteine residues were alkylated with 10mM iodoacetamide. The solution was diluted 1:4 and digested with 1μg of trypsin at 37°C overnight. The digestion was terminated by adding formic acid to 2%. Mass spectrometry and data analysis were performed in the following workflow: the protein digest was analyzed by an 11-step MudPIT. A cycle of one full-scan mass spectrum (400-1800 m/z) at a resolution of 60,000 followed by 20 data dependent MS/MS spectra at a 35% normalized collision energy was repeated continuously throughout each step of the multidimensional separation. Protein identification was performed with the Integrated Proteomics Pipeline-IP2 (Integrated Proteomics Applications, Inc., San Diego, CA. http://www.integratedproteomics.com/) using ProLuCID and DTASelect2. The tandem mass spectra were searched against the EBI IPI mouse protein database. In order to accurately estimate peptide probabilities and false discovery rates, we used a target/decoy database containing the reversed sequences of all the proteins appended to the target database. The protein false discovery rates were controlled below 1% for each sample analysis. The protein lists depicted in Fig 3D were filtered to ensure they exhibit the same IRE1α-dependent trend as measured by mRNA-Seq. Full results are provided in Data S2.

**Masson’s trichrome stain**

Collagen fibers were detected as described:

<http://www.ihcworld.com/_protocols/special_stains/masson_trichrome.htm>

**cDNA synthesis for quantitative real time PCR (qRT-PCR)**

BioRad’s iScript cDNA synthesis was performed using blended oligo-dT and random primers on total RNA from islets or cells. RNA was purified using the Stratagene column-based RNA purification kit. The BioRad sybr green based detection system was utilized to calculate ^ΔΔ^Ct folds relative to the housekeeping genes β-actin and/or 18S rRNA. Primer efficiencies were also determined to be ≥90% by cDNA dilution. qRT-PCR primer sequences were obtained from the Harvard Primer Bank; <http://pga.mgh.harvard.edu/primerbank/>.

**Glucose tolerance tests and insulin and proinsulin measurements**

A 20% by weight to volume of glucose in water solution was injected intraperitoneally at a final dose of 2g glucose/1Kg body weight and then blood glucose was recorded by tail snip glucometer readings over time. When blood glucose readings exceeded the range of the glucometer, 10µl blood was diluted with 20µl PBS with 1mM EDTA before reading. CrystalChem (Cat.90060) and Alpco (Cat.80-PINMS-EO1) insulin and proinsulin ELISAs were used on 50mg/ml pancreas from acid ethanol extracts after determining the proper dilution (1:200) in sample resuspension buffer. For *in vivo* secretion, measurements were taken on 30μl serum and for the proinsulin ELISA lower standards were prepared and the colorimetric step was extended to one hour rather than thirty minutes.

**Immunofluorescence and Immunohistochemical microscopy**

Paraffin embedded pancreas sections were deparaffinized, rehydrated then boiled for 10 min in citrate buffer for antigen retrieval. A solution of 1% BSA, 1% FBS in TBST was used to block and wash slides and for antibody dilutions: anti-insulin, pro-insulin and KDEL (1:500), glucagon (1:100), GLUT2 (1:200), and secondary antibodies (1:1500). Primary incubations were 24 hours at 4ºC and secondary incubations were for 3 hours at 25ºC. Images were acquired identically using a minimum of 6 panels per mouse and 6 mice per genotype. Quantitation of fluorescent signals was performed using the Cell Profiler software and statistical analysis by student t-test derived p-values. Custom macros within Aperio’s ScanScope version 8 <https://scanscope08/Welcome.php> were designed to detect the brown color formed by the secondary precipitate-forming reactions of the anti-nitrotyrosine and the anti-tenascin C while a macros detecting blue collagen stain was generated for the Masson’s trichrome stain. Examples of the custom macros signal detection for nitrotyrosine are presented in Fig 4C. In all cases a negative control containing no primary antibody with only the secondary was used to determine non-specific reactivity. Islets were circled and analyzed by the macros specifically.

**Antibodies**

Proinsulin and insulin antibodies for immunofluorescence, immunoprecipitation and Western blotting were from HyTest cat. # 2PR8 (CCL-10) and Sigma cat. # K36AC10. The antibody for KDEL (BIP and GRP94) was from Enzo cat. # ADI-SPA-827. Anti-p-IRE1α is a rabbit antibody specific for the pS724 epitope made in-lab. RPS9 and OS9 were goat antibodies from Santa Cruz cat. #s: sc162106 and 168854. Rabbit anti-GLUT2 was from Santa Cruz cat. # sc9117. Mouse anti-tubulin was from Sigma cat. # T9026. Mouse monoclonal anti-β-actin was from Sigma cat. # A5316. The tenascin C monoclonal rabbit antibody was from Cell Signaling cat. # 12221.

**Metabolic proinsulin synthesis**

Isolated islets (50/sample) were deprived of Cys/Met for five min then incubated in RPMI with the indicated glucose concentration lacking Cys/Met and containing 200µCi/ml [^35^S]-Cys/Met for thirty minutes. Samples were lysed in 200µl 2%SDS, 150mM NaCl with protease and phosphatase inhibitors then 15µl pre-I.P. lysate was removed for trichloroacetic acid precipitation (cat. #88802 Pierce Co.) to normalize loading onto a 12% Bis-Tris gel (BioRad cat. #345-0119) for SDS-PAGE. Gels were plasticized with PEG-1500, dried and quantified by phosphor-imaging using Image-Quant software.

- **Bioinformatics analysis and gene ontology**
- IRE1α- and glucose- dependent mRNAs were identified by limiting the original 22k mRNAs detected to those with ≥1.5 +/- fold changes relative to the 6mM *WT* control islets with p-values ≤0.05 were then loaded into the 4-way Venn diagram generator: <http://www.pangloss.com/seidel/Protocols/venn4.cgi>**.** Gene ontology was determined using the DAVID bioinformatics database <http://david.abcc.ncifcrf.gov/> and ConceptGen: <http://conceptgen.ncibi.org/core/conceptGen/index.jsp>.
